# Using spatial genetics to quantify mosquito dispersal for control programs

**DOI:** 10.1186/s12915-020-00841-0

**Published:** 2020-08-20

**Authors:** Igor Filipović, Hapuarachchige Chanditha Hapuarachchi, Wei-Ping Tien, Muhammad Aliff Bin Abdul Razak, Caleb Lee, Cheong Huat Tan, Gregor J. Devine, Gordana Rašić

**Affiliations:** 1 Mosquito Control Laboratory, QIMR Berghofer Medical Research Institute, 300 Herston Road, Herston, QLD 4006 Australia; 2 Environmental Health Institute, National Environment Agency, 11, Biopolis Way, #06-05-08, Singapore, 138667 Singapore

**Keywords:** Mosquito dispersal, Genome-wide SNPs, Close kin, Dispersal kernel, IBD, Spatial autocorrelation

## Abstract

**Background:**

Hundreds of millions of people get a mosquito-borne disease every year and nearly one million die. Transmission of these infections is primarily tackled through the control of mosquito vectors. The accurate quantification of mosquito dispersal is critical for the design and optimization of vector control programs, yet the measurement of dispersal using traditional mark-release-recapture (MRR) methods is logistically challenging and often unrepresentative of an insect’s true behavior. Using *Aedes aegypti* (a major arboviral vector) as a model and two study sites in Singapore, we show how mosquito dispersal can be characterized by the spatial analyses of genetic relatedness among individuals sampled over a short time span without interruption of their natural behaviors.

**Results:**

Using simple oviposition traps, we captured adult female *Ae. aegypti* across high-rise apartment blocks and genotyped them using genome-wide SNP markers. We developed a methodology that produces a dispersal kernel for distance which results from one generation of successful breeding (effective dispersal), using the distance separating full siblings and 2nd- and 3rd-degree relatives (close kin). The estimated dispersal distance kernel was exponential (Laplacian), with a mean dispersal distance (and dispersal kernel spread σ) of 45.2 m (95% CI 39.7–51.3 m), and 10% probability of a dispersal > 100 m (95% CI 92–117 m). Our genetically derived estimates matched the parametrized dispersal kernels from previous MRR experiments. If few close kin are captured, a conventional genetic isolation-by-distance analysis can be used, as it can produce σ estimates congruent with the close-kin method if effective population density is accurately estimated. Genetic patch size, estimated by spatial autocorrelation analysis, reflects the spatial extent of the dispersal kernel “tail” that influences, for example, the critical radii of release zones and the speed of *Wolbachia* spread in mosquito replacement programs.

**Conclusions:**

We demonstrate that spatial genetics can provide a robust characterization of mosquito dispersal. With the decreasing cost of next-generation sequencing, the production of spatial genetic data is increasingly accessible. Given the challenges of conventional MRR methods, and the importance of quantified dispersal in operational vector control decisions, we recommend genetic-based dispersal characterization as the more desirable means of parameterization.

## Background

Mosquitoes’ ability to carry and transmit human pathogens (malaria and filarial parasites, arboviruses) causes hundreds of millions of infections and nearly one million fatalities every year [1]. Both prevention and mitigation of many mosquito-borne disease outbreaks are primarily reliant on the control of mosquito vectors. Most of these interventions are designed to impact mosquito abundance or daily survival by targeting immature and adult stages. In the case of the major arbovirus vector *Aedes aegypti*, an urban-dwelling container-breeding mosquito, conventional control approaches include removal and treatment of larval habitats, as well as elimination of adults through insecticide application (indoor-residual and space spraying) and lethal trapping [2]. New biocontrol strategies undergoing field evaluations include RIDL^R^ and *Wolbachia-*based population replacement and suppression [3–5].

Defining the optimal area for treatment or mosquito release is one of the key considerations when implementing a public health intervention or designing a field-trial for a new control approach. For example, to contain the spread of dengue virus during an outbreak, focal insecticide-based control of *Ae. aegypti* adults is typically conducted at and around the main and secondary residences of dengue cases. The radius of the area to be treated is informed by the average dispersal distance of potentially infected female *Ae. aegypti* [6]. Understanding the ability of released sterile male mosquitoes to disperse and mate in an area being targeted by a suppression strategy is essential for predicting the required release pattern [7]. Additionally, sustained suppression in a target zone is difficult if a surrounding buffer zone is too small to prevent immigration by gravid wild-type females from neighboring areas. Similarly, stable introduction of a virus-blocking *Wolbachia* may fail if the release area of *Wolbachia-*infected *Ae. aegypti* is too small and too vulnerable to immigration by wild-type mosquitoes [8]. For the emerging genetic-based control approaches such as gene drive systems [9], well-characterized mosquito dispersal is crucial for addressing the biosafety concerns around the systems’ confineability and reversibility in the field [10, 11].

Quantifying mosquito dispersal of both wild-type and introduced mosquito strains in any given landscape is, therefore, critical for the considerations of the size of the treatment area and the surrounding buffer zones. Those considerations complement practical operational deliberations of the availability of human and economic resources for implementation and the sample sizes required to capture epidemiological endpoints [12, 13].

Mosquito dispersal characteristics have been typically studied using conventional mark-release-recapture (MRR) experiments utilizing powders and paints on trapped or laboratory-reared adult mosquitoes [14]. Location of the recaptured marked insects relative to their release point is typically used to estimate the mean distance traveled (MDT), and the distance within which 50% or 90% of mosquitoes are expected to disperse (FR50 and FR90, respectively). Fewer MRR studies have incorporated the dispersal kernel theory to estimate the distribution of dispersal distances over the whole flight range [7, 15, 16]. MRR experiments in *Ae. aegypti* have reported the mean dispersal distance to range from tens to hundreds of meters [14], and this variation points to a need to characterize dispersal locally so that the optimal control can be deployed in a given landscape. However, MRR experiments are operationally demanding, and the rearing and marking procedure can alter mosquito fitness and movement behavior in the field [17]. Additionally, the release of biting vector-competent females might pose an unacceptable risk of increased pathogen transmission in endemic areas.

Here we show how information on mosquito dispersal characteristics can be obtained from the spatial patterns of genetic distance and relatedness among sampled individuals, providing an alternative to the MRR experiments for informing the mosquito control programs. In contrast to conventional MRR approaches that require insect trapping or rearing, followed by mark, release, and recapture, the genetic approach requires only insect capture, utilizing the information from genetic markers and spatial location of individuals sampled continuously across an area over a limited time span and without manipulation or interruption of their natural behaviors.

In social insects like bumblebees, queen dispersal distance has been estimated by comparing the locations of workers (sampled in summer) and queens (sampled in the following spring) that were identified as sisters through sibling reconstruction analysis with genetic markers [18]. Inferences about mosquito ovipositing behavior have also been made using the genetic reconstruction of sibling groups, where the distance between full siblings sampled from different larval sites directly reflects mother’s movement distance during her skip-oviposition [19–21]. Here we show that the distance separating not only full siblings (1st-degree relatives), but also 2nd- and 3rd-degree relatives (close kin), can be used to estimate the dispersal distance over one generation of successful breeding (i.e., effective dispersal distance) in insects like *Ae. aegypti*.

Our newly developed method decomposes the observed separation distances between close kin (sampled as breeding adults) to generate the distribution of potential effective dispersal distances and to parametrize the dispersal distance kernel. This dispersal kernel provides the density of probability that a dispersal event ends at a given distance away from the source, regardless of the direction. Importantly, it refers to the dispersal distance achieved over one generation of successful reproduction, such as distance between the birthplace and the ovipositing place of a female (i.e., distance between the ovipositing place of a mother and a daughter). We demonstrate the robustness of the method to produce dispersal kernel parameters consistent among different subsets of data (with one or multiple kinship categories) and congruent with the estimates of dispersal characteristics from the previously published MRR experiments in *Ae. aegypti*.

When few close kin are captured, the conventional genetic analysis of isolation-by-distance (IBD) between unrelated individuals can be used to estimate the spread of the dispersal kernel from the slope of the IBD relationship and the effective density, and we show that its results can be congruent with the new close-kin method. Finally, we show that the estimated genetic patch size (area of high local dispersal and breeding) from the spatial autocorrelation analysis reflects the spatial extent of the effective dispersal distance kernel’s “tail” that cannot be ascertained with IBD analysis alone.

We analyzed the genotyped and geo-referenced *Aedes aegypti* individuals collected in two densely populated areas of Singapore with a homogeneous distribution of high-rise apartment buildings. *Aedes aegypti* is the primary vector of dengue virus in Singapore that, despite having a low *Aedes* house index (2%) and an extensive vector surveillance and control program [22], continues to experience regular dengue outbreaks [23, 24]. This dataset offered an opportunity to characterize *Ae. aegypti* dispersal in a highly urbanized landscape with a prominent vertical dimension, but the spatio-genetic analytical approach (the new close-kin method, the conventional IBD and spatial autocorrelation analyses) can be applied across different landscapes and vector species.

## Results

### Entomological sampling

**Fig. 1:**
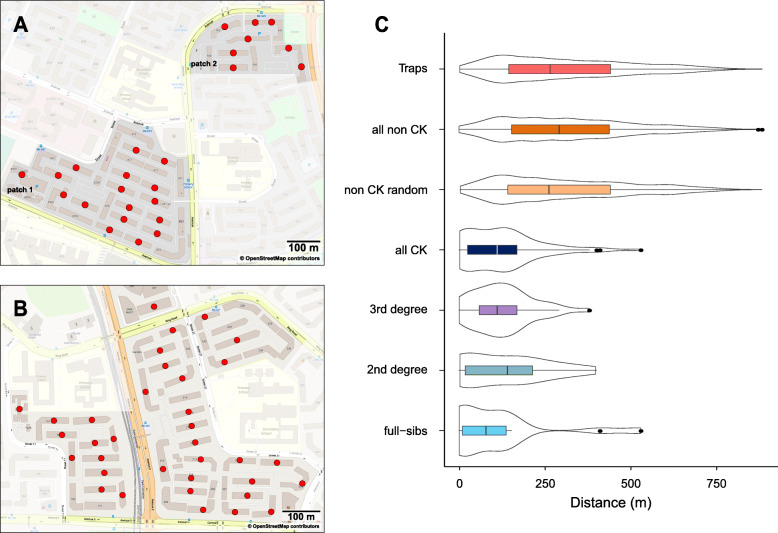
Sampling locations and density distributions of observed separation distances. Red dots indicate the vertical trapping locations in apartment buildings in Tampines (**a**) and Yishun (**b**). Horizontal violin plots (**c**) show the density distribution of separation distance for full siblings, 2nd-degree relatives, 3rd-degree relatives, all close kin combined (“all CK”), non-close kin (“all non CK”), null distribution (“non CK random”), and traps. The box within each violin plot shows the interquartile range and the location of the median

Adult *Aedes aegypti* females were collected using the simple sticky traps (Gravitraps) [25] that were positioned for vertical sampling in high-rise apartment buildings (ground level, 4th–5th floors, ≥ 9th floor) in two public housing estates: Tampines and Yishun (Fig. 1a, b). In both sites, the median number of traps per week was six (range 5–8 in Tampines, 2–9 in Yishun). The median number of trapped females per building per week was four in Tampines (range 1–17) and two in Yishun (range 1–4). Out of 107 sequenced females from Tampines, 88% were collected between May 16 and 20, 2018 (week 20), and 12% between May 21 and 22 (week 21), from a total of 128 traps positioned across 25 buildings (Fig. 1a). Out of 108 sequenced females from Yishun, 79.6% were collected between April 22 and 27 (week 17), 13.9% between May 1 and 2 (week 18), 1.9% on May 8, and 4.6% on May 15 (week 20). These were collected from a total of 224 traps across 35 buildings in Yishun (Fig. 1b). Details on the weekly number of traps and collected females per building (for weeks 1–22 in 2018) are found in Additional file 1: Tables S1-S4.

### Spatial distribution of close-kin pairs

Reduced representation genome sequences for individual *Ae. aegypti* females were obtained using the double-digest RAD sequencing [26] following the library preparation protocol by Rašić et al. [27]. Genotype likelihoods at 24–60 k genome-wide SNP positions (median = 57,947) were used to estimate the relationship between all pairs of individuals, using the combinations of three statistics (R0, R1, and KING-robust) [28, 29] to determine the kinship category as parent-offspring, full siblings, 2nd-degree relatives (half siblings, avuncular, grandparent-grandoffspring), 3rd-degree relatives (first cousins), and non-close kin.

In the total dataset, we detected 76 close-kin pairs: 19 full-sibling pairs, 18 pairs of 2nd-degree relatives, and 39 pairs of 3rd-degree relatives. We did not detect any parent-offspring (*po*) pairs, and 26.3% (20/76) of close-kin pairs were found on the same floor or 4–5 floors apart (19/20 pairs). Nearly half (47%) of all detected full-sibling pairs were caught within a building, in comparison to 27.8% of all 2nd-degree relatives and 15.4% of all 3rd-degree relatives. The members of each close-kin pair were sampled within 8 days (median = 1 day apart).

The median pairwise distance between close kin (CK) was 111.6 m (90th percentile = 264 m), with the maximum distance of 531 m, giving a positively skewed distribution of distances (skewness = 1.24, kurtosis = 1.90; Fig. 1c “all CK”). To compare this distribution to a null distribution (for randomly spaced individuals across the matrix of traps), we created 100 random subsamples (with replacement) of recorded pairwise distances between individuals not assigned to any kinship category (non-close kin). The null distribution (Fig. 1c “non CK random”) had a median of 263.6 m and was significantly different from the distribution of pairwise distances between close-kin (the permutation test of equality of two distribution densities *p* < 0.001).

The median distance between full siblings was 79.5 m, but we also detected two instances of pairwise distance > 400 m (Fig. 1c, Additional file 2: Fig. S1-S2), indicating the “long-tailed” dispersal kernel. The median distance between 2nd- and 3rd-degree relatives was 141.3 m and 112 m, respectively, and we did not detect outliers beyond 402 m for these two kinship categories (Fig. 1c). This suggests the spatial extent and configuration of our sampling could have missed some rare long-range dispersal events that accumulated a substantial separation between close kin over more than two generations (more so for 3rd-degree relatives than for 2nd-degree relatives). Additionally, some “outlier” 3rd-degree relatives could have been incorrectly assigned to a non-close-kin category, given that the kinship estimation is likely downward biased due to local population structure [30, 31] (positive spatial autocorrelation, isolation-by-distance; see below).

### Dispersal kernel parametrization from close-kin data

A distance separating close kin is a result of dispersal and successful breeding over multiple generations, and here we show how it can be used to infer a dispersal distance achieved over one generation of successful breeding. This distance is also known as the “effective dispersal distance” [32] and can be defined as a distance between the birthplace and the ovipositing place of a female (or a distance between the ovipositing place of a mother and a daughter). Every close-kin category contains information about the number of possible dispersal and breeding events over one generation that can be represented as a set. This set of numbers is used to divide the observed spatial distance between each close-kin pair to create a set of possible effective dispersal distances. By combining the values from all pairwise sets into one dataset, we can characterize the resulting distribution of possible effective dispersal distances. This is done by finding the “best-fitting” function (e.g., exponential, Weibull, log-normal) to generate a probability density function (*pdf*) of effective dispersal distance, i.e., effective dispersal distance kernel. A detailed description of the method is found in the “Methods” section.

**Fig. 2:**
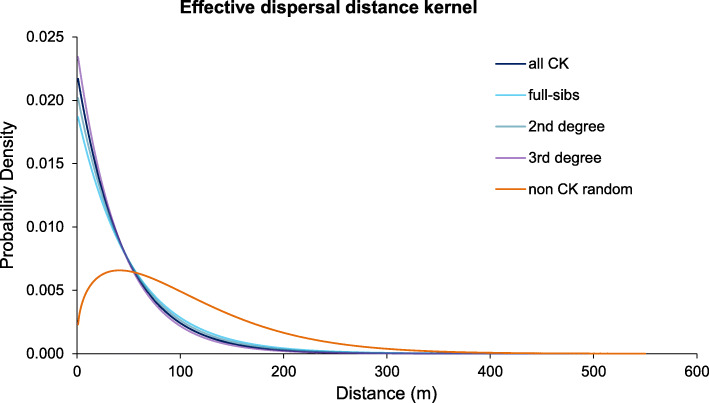
Effective dispersal distance kernel estimated from the close-kin data. The inferred *pdf*s are highly congruent among separate datasets (full sibling, 2nd- and 3rd-degree relatives) and the combined dataset (“all CK”), and are significantly different from the randomly subsampled non-close kin dataset (“non CK random”) that represents the null distribution of distances for randomly spaced individuals across the matrix of traps

The “goodness of the fit” criteria (AIC, BIC, the Kolmogorov-Smirnov statistic [33], Table 1) and the Q-Q plot (Additional file 2: Fig. S3) indicated that the distribution of possible effective dispersal distance derived from the close-kin data in Tampines and Yishun is well described by the Weibull and negative exponential (Laplacian) distribution. Given that the estimated Weibull shape parameter (*k*) was close to 1 (median 1.11, 95% CI 1.01–1.22), the Weibull distribution can be reduced to an exponential distribution with the estimated rate parameter *λ* = 0.022 (95% CI 0.020–0.025) (Table 1). This rate parameter for the combined dataset (“all CK”) was highly congruent with the estimates from separate CK categories: full siblings *λ* = 0.019 (95% CI 0.014–0.027), 2nd-degree relatives *λ* = 0.021 (95% CI 0.016–0.027), 3rd-degree relatives *λ* = 0.024 (95% CI 0.020–0.028) (Fig. 2). Both the mean and standard deviation (σ) of the exponentially distributed effective dispersal distance are 1/*λ* = 45.2 m (95% CI 39.7–51.3 m), and the inferred dispersal kernel gives the 95% probability of effective dispersal distance up to 136 m (95% CI 120–152 m).

**Table 1:** Goodness-of-fit statistic and criteria and parameter estimates from the distribution fitting analysis for close-kin data

**Goodness-of-fit test**	**Negative exponential (Laplacian)**	**Weibull**	**log-normal**
KS	0.084	0.057	0.125
AIC	2387.913	2386.055	2429.829
BIC	2391.427	2393.082	2436.856
**Parameter**	*λ*	Shape	Meanlog
Median (95% CI)	0.022 (0.020–0.025)	1.108 (1.011–1.222)	3.322 (3.170–3.462)
		Scale	sdlog
	–	46.939 (41.770–52.445)	1.162 (1.055–1.252)

A lower value of the statistic (the Kolmogorov-Smirnov statistic, KS) or the criterion (AIC, BIC) indicates a better fit. The parameter median and 95% CI were generated with 1000 bootstraps

We repeated the same analytical procedure for the null distribution (random sampling of pairwise distances between non-close kin). The null distribution produced a distance kernel with the Weibull shape *k* of 1.35 (95% CI 1.34–1.36) and Weibull scale *λ* of 111.63 (95% CI 110.50–112.70) (Fig. 2) and was significantly different from the close-kin-derived kernel (permutation test for equality of densities *p* < 0.001).

### Dispersal kernel spread from the isolation-by-distance (IBD) analysis

When the probability of dispersal declines with distance, a positive correlation between genetic and geographic distances between individuals is expected, and this relationship is known as isolation-by-distance (IBD) [34, 35]. IBD is best illustrated by the regression of pairwise genetic distances onto geographic distances between individuals [34]. Applying the IBD theory, we estimated the standard deviation of the dispersal kernel (σ), also known as the dispersal kernel spread, using the slope of the IBD relationship and the effective population density [36]. Three different genetic distance measures (PCA-based [37], Rousset *â* [36], Loiselle’s kinship [38]) gave highly congruent results in the IBD analysis (Additional file 1: Table S5), and we focus on the results with the PCA genetic distance in the main text.

**Fig. 3:**
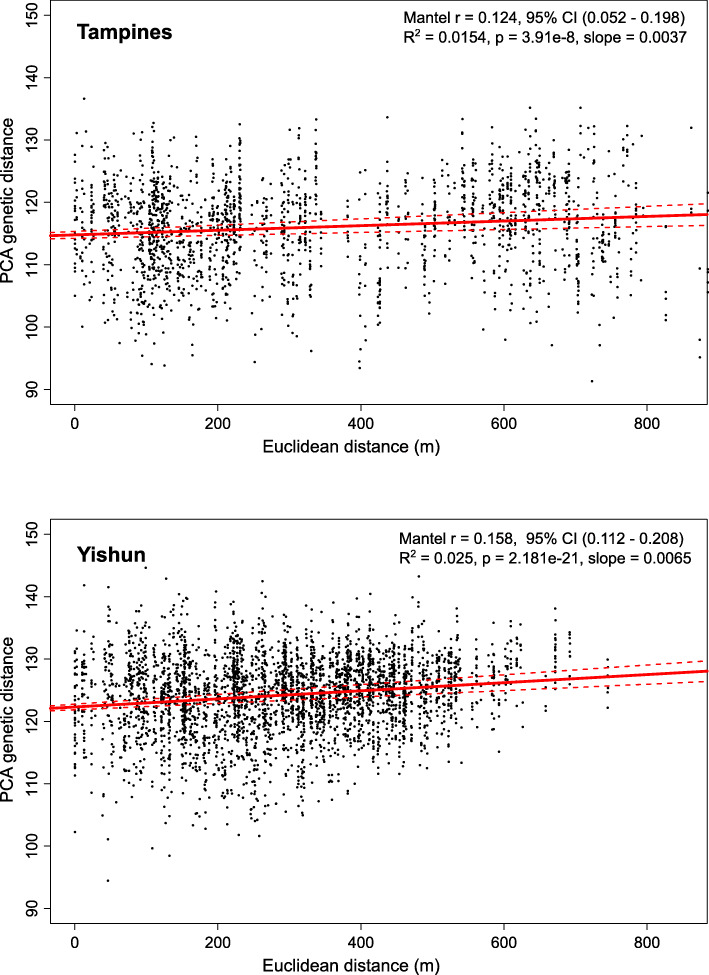
Isolation-by-distance analysis on non-close kin data from Tampines and Yishun. Mantel test and linear regression were applied to the matrices of PCA genetic distance and linear geographic distance between pairs of individuals in Tampines (upper) and Yishun (lower), with close kin removed from both datasets. The red line shows regression with 95% CI (dashed lines)

Significant correlation between PCA genetic distance and linear geographic distance (IBD) was detected between non-close kin in Tampines (Mantel *r* = 0.124, 95% CI 0.052–0.198; *R*^*2*^ = 0.015, *p* = 3.91 × 10^−8^) and Yishun (Mantel *r* = 0.158, 95% CI 0.112–0.208; *R*^*2*^ = 0.024, *p* = 2.18 × 10^−21^) (Fig. 3). Because genetic distance between individuals can also be driven by the time of sampling (temporal distance), we confirmed that correlation between genetic and geographic distance (IBD) remained significant after the effect of temporal distance was removed (Partial Mantel test *p* ≤ 0.01 for Tampines and Yishun; Additional file 1: Table S6). Absence of temporal structuring in our data was not surprising, given that we trapped ≥80% of analyzed individuals within 1 week (> 93% within 2 weeks) in each site.

The estimated IBD slope *b* was 0.0037 (95% CI 0.0024–0.0050) for the Tampines data, and 0.0065 (95% CI 0.0051–0.0079) for the Yishun dataset (Table 2). The estimated effective population density *D* varied from 0.0014 to 0.0074 for Tampines and from 0.0014 to 0.0063 for Yishun, depending on the method for effective population size estimation (method 1 PWoP [39], method 2 LDN_e_ [40]), or the entomological survey data (method 3 Gravitrap) (Table 2).

**Table 2:** IBD-based estimates of the dispersal kernel spread (σ)

***b***		***N***_***e***_	***D***	**σ**
**TAMPINES**				
0.0037 (0.0024–0.0050)	Method 1 (PWoP)	863 (863–1112)	0.0074 (0.0074–0.0095)	54.1 m (40.8–67.7 m)
	Method 2 (LDN_e_)	167 (93–619)	0.0014 (0.0007–0.0053)	122.9 m (54.7–206.2 m)
	Method 3 (Gravitrap)	–	0.0048 (0.0030–0.0066)	66.8 m (48.8–105.6 m)
**YISHUN**				
0.0065 (0.0052–0.0079)	Method 1 (PWoP)	1185 (1176–1346)	0.0063 (0.0063–0.0072)	44 m (37.6–49.7 m)
	Method 2 (LDN_e_)	258 (200–357)	0.0014 (0.0011–0.0019)	94.4 m (73–120.6 m)
	Method 3 (Gravitrap)	–	0.0022 (0.0015–0.0030)	74.4 m (57.9–104.9 m)

The mean (95% CI) for IBD slope (*b*)*,* effective population size (*N*_*e*_), effective density (*D*) estimated using the methods 1–3, and the dispersal kernel spread (σ) for *Aedes aegypti* data from Tampines and Yishun

**Fig. 4:**
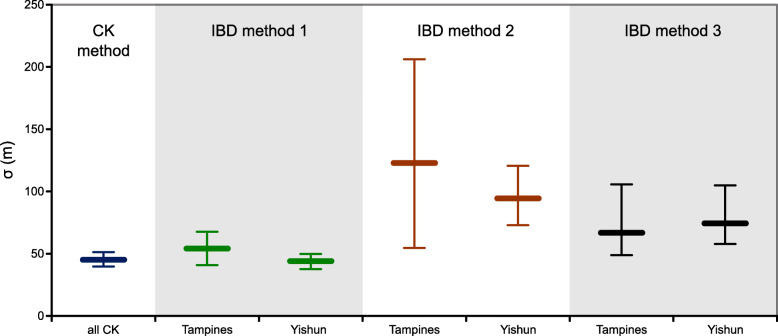
The dispersal kernel spread (σ) estimated from the close-kin data and IBD analysis. Sigma (σ) and its 95% CI are plotted for the combined close-kin data (CK method) and PCA-based IBD analysis for Tampines and Yishun (with effective density estimates from methods 1–3)

Taking into account the uncertainty of both parameter estimates (95% CI for *b* and *D*), the estimated effective dispersal kernel spread (σ) for Tampines was 54.1 m (40.8–67.7 m, method 1), 122.3 m (54.7–206.2 m, method 2), and 66.8 m (48.8–105.6 m, method 3). For Yishun, the estimated σ was 44 m (37.6–49.7 m, method 1), 94 m (73–120.6 m, method 2), and 74.4 m (57.9–104.9 m, method 3) (Table 2, Fig. 4). It is worth noting a good overlap between the effective density estimates from the genetic data and the entomological data from the gravitraps (method 3) that preferentially target the ovipositing females (Fig. 4).

Applying the exponential dispersal kernel (found to fit the close-kin data), where σ represents both standard deviation and the mean, the estimates of σ from the IBD analysis are equivalent to the mean effective dispersal distance and can be used to parametrize the *pdf* with 1/σ = *λ*, assuming isotropic dispersal in two dimensions [32]. Our results indicate that the dispersal kernel parameter (σ) estimated from the close-kin data and indirectly through the IBD analysis can yield similar results (Table 2, Fig. 4).

### Spatial autocorrelation analysis—genetic patch size

**Fig. 5:**
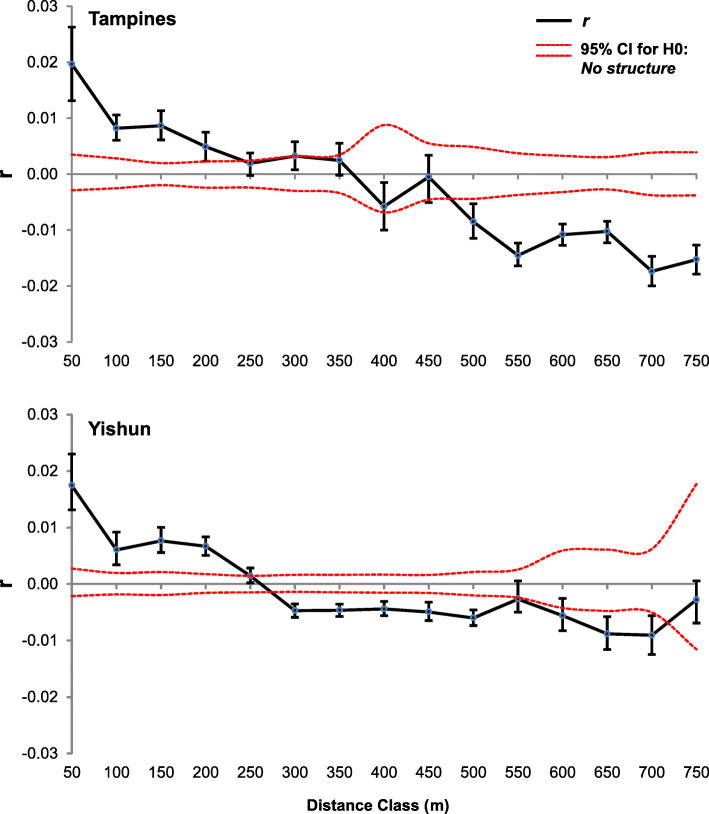
Spatial genetic autocorrelation in Tampines and Yishun. The ending point of a distance class is on the *x*-axis, and spatial autocorrelation coefficient (*r*) of genotypes in Tampines (107 individuals) and Yishun (108 individuals) is on the *y*-axis. Two dashed lines along the *x*-axis are the permutated 95% CI of autocorrelations under the null hypothesis of a random distribution of genotypes in space. Vertical lines are the bootstrapped 95% CIs with the mean genetic autocorrelation

Under spatially limited dispersal and breeding, the population is expected to develop a patchy distribution of genotypes, with positive spatial autocorrelation declining with distance [41, 42]. We detected significantly positive spatial autocorrelation at distances up to 200 m, with the highest correlation coefficient within the first 50 m, in both Tampines and Yishun (Fig. 5). This indicates that individuals found up to 200 m from each other are more genetically similar than if randomly sampled across 750 m, with the highest genetic similarity (relatedness) within 50 m from each other. Significantly negative autocorrelation was detected at 300 m in Yishun and 500 m in Tampines, putting the point at which the correlogram curve crosses the *x*-axis (*x*-intercept) between 200 and 300 m in Yishun and between 200 and 500 m in Tampines (Fig. 5). For a squared sampling area, the x-intercept closely approximates the length of one side of a “genetic patch” [43] or an area of high level of localized dispersal and breeding [41, 42]. In Tampines and Yishun, the size of the genetic patch is estimated to be at least 200 × 200 m.

## Discussion

Here we show how the analyses of spatial genetic patterns can be used to characterize the effective dispersal of a mosquito like *Ae. aegypti*, and we discuss the utility of this approach in an operational context.

Our newly developed method allows for the parametrization of the effective dispersal distance kernel, as it decomposes the observed distances between close kin to generate the distribution of potential effective dispersal distances (achieved over one generation of successful reproduction). It gives probabilities of dispersal distances in any direction, referred to as the “dispersal distance kernel” (*k*_D_(*r*)) [44], and it should not be confused with the “dispersal location kernel” (*k*_L_(*r*)) that gives probabilities for the end locations of dispersers relative to the source locations [44]. The location kernel can be derived from the distance kernel and vice versa, given their relation: *k*_D_(*r*) = 2π*rk*_L_(*r*) in a two-dimensional habitat [44], and *k*_D_(*r*) = 4π*r*^2^*k*_L_(*r*) in a three-dimensional habitat.

Our method has some obvious parallels to the work of Jasper et al. [20] that also utilizes separation distance between close kin to infer dispersal distance over one generation, and the protocol by Rašić et al. [27] to generate genome-wide SNP data in *Ae. aegypti* collected from high-rise buildings. However, there are weaknesses in the method by Jasper et al. [20] that are not present in our method. First, their underlying assumption is Gaussian dispersal (normal distribution of dispersal distance [35]), even though the observed dispersal kernel in mosquitoes and other insects tends to be more leptokurtic with a higher probability of short- and long-distance dispersal [7, 44, 45]. Our method allows the inference of a best-fitting distribution (e.g., negative exponential, Weibull, log-normal, etc., Fig. 2) when parameterizing the dispersal kernel. Many modeling studies have shown that processes such as population spread behave differently when long-tailed dispersal distributions are used instead of Gaussian [46]. For example, an incorrect assumption of Gaussian dispersal can have operational impact on a mosquito control campaign like the *Wolbachia-*based replacement, because it would cause a perception that *Wolbachia* spreads through a population more slowly than expected (by as much as 40%, depending on the true shape of non-Gaussian dispersal kernel) [47]. Second, the Jasper et al. method [20] produces very wide confidence intervals for the estimated dispersal kernel spread (95% CI for σ = 23–93 m), while our method achieves much greater precision (95% CI for σ = 40–51 m). Third, Jasper et al. [20] produce a non-trivial number of cases where σ has a value of an imaginary number (square root of a negative number), and this raises concerns about the fundamental properties of their method.

Using the close-kin data from the Tampines and Yishun districts in Singapore, our close-kin method produced the negative exponential (Laplacian) kernel that gives 50% probability (kernel median) of effective dispersal occurring within 32 m. This indicates that, in this landscape, we can expect half of the successfully breeding individuals to stay within the high-rise building where they emerged or move to the adjacent building. The mean effective dispersal distance (and dispersal kernel spread σ) was estimated at 45.2 m (95% CI 39.7–51.3 m), with a 10% probability of a dispersal distance greater than 100 m (95% CI 92–117 m). Our genetic-based estimates match the parametrized dispersal kernel from mark-release-recapture (MRR) experiments performed in Brazil and Malaysia with *Ae. aegypti* males from a genetically engineered line OX513A [7]. Namely, their dispersal kernel gave estimates of a high level of dispersal to up 33 m, a mean distance traveled of 52.8 m (95% CI 49.9–56.8 m) in Brazil and 58 m (95% CI 52.1–71 m) in Malaysia, with 10% of dispersers moving > 105.7 m (95% CI 86.5–141.1 m) [7]. Moreover, globally collated MRR experimental data for *Ae. aegypti* [14] produced an exponential kernel with σ = 54.1 m [10]. Given that we used a trapping system that preferentially catches ovipositing females, our close-kin-based kernel is more influenced by female dispersal. However, a high congruence with the MMR-based kernel for males from similar landscapes suggests similar dispersal in both sexes*,* as well as the robustness of spatial genetic patterns in reflecting the dispersal characteristics of this mosquito.

The IBD pattern reflects effective dispersal of both sexes averaged out over many generations, and our σ estimates from the IBD analysis indicate that they can match the short-term measures derived from the close-kin analysis. Given that the close-kin method requires more intensive sampling in order to capture enough close-kin pairs for the reliable kernel parametrization, the use of IBD analysis as an alternative is appealing, particularly under budgetary limitations for genome-wide genotyping. However, IBD-based estimation of σ requires accurate estimation of effective population size, which it is not easily achievable [48, 49], and the uncertainty about this parameter has more impact than the uncertainty in the IBD slope [50]. The highest congruence between the IBD and the close-kin analysis was obtained using the *N*_*e*_ estimates from the PWoP method [39] (method 1, Table 2), which gave σ of 54.1 m (95% CI 40.8–67.7 m) for Tampines and 44 m (95% CI 37.6–49.7 m) for Yishun. The second best match was obtained using the entomological effective density estimate (method 3) that gave σ of 66.8 m (95% CI 48.8–105.6 m) for Tampines and 74.4 m (95% CI 57.9–104.9 m) for Yishun. This is interesting, as it suggests that the gravitrap data could be used as an entomological proxy for the effective population size, complementing the genetic-based estimation of this population parameter. The linkage disequilibrium-based *N*_*e*_ estimate [40] (method 2) produced the widest range of σ values (Tampines 54.7–206.2 m, Yishun 73–120.6 m) (Fig. 4). This LD-based estimator is expected to be less precise than the PWoP estimator [39] and also downward biased when IBD is present in a continuously distributed population, because it reflects the local genetic neighborhood size (*N*_*b,*_ [35]) rather than the effective size of the global population (*N*_*e*_) [51]. We observed this trend in *Ae. aegypti* from Tampines and Yishun (with LD *N*_*e*_ being similar to *N*_*b*_; Additional file 1: Table S5). In addition to the limitations related to *N*_*e*_ estimation, the IBD method assumes long-term stability of mosquito dispersal patterns and abundance, making it is a meaningful alternative to the close-kin method in populations that do not experience strong seasonal fluctuations, landscape alterations, intensive control campaigns, etc.

In Tampines and Yishun, that are largely homogeneous landscapes with multi-storey apartment buildings, 47% of all detected full siblings were found on the same floor or 4–5 floors apart, and the inferred dispersal kernel predicts high level of effective dispersal within a building or between adjacent buildings. This agrees with a previous study in Singapore where *Ae. aegypti* females marked with rubidium via spiked blood meal were released from middle floors and moved readily towards the top or bottom of multi-storey buildings in search of oviposition sites [52]. In Trinidad, significantly more eggs were collected in ovitraps 13–24 m above ground level than at any other elevation [53], again suggesting that vertical movement is common.

Given that high-rise apartment blocks can provide an abundance of hosts, oviposition, and resting sites, the tendency of *Ae. aegypti* to remain close to the birthplace is not unexpected. The tail of the dispersal kernel, however, provides insight into rarer long-range dispersal events that are consequential for the control strategies that rely on the releases of modified mosquitoes for population suppression or replacement. For example, the *Wolbachia* spread is expected to be slower in *Ae. aegypti* populations with longer-tail dispersal kernels, but this dispersal pattern also allows the initiation of the *Wolbachia* spread with smaller local releases [47]. In a population with a dispersal kernel like in Tampines and Yishun, theoretical approximation [47] predicts that the spread of *Wolbachia* (with a fitness cost equivalent to *w*Mel strain) could be initiated if *Wolbachia-*infected *Ae. aegypti* are released in an area with a radius of at least 100–130 m (2.51σ for Laplacian kernel [47]). The diameter of this release area (200–260 m) matches the end of the effective dispersal kernel’s “tail” (99th percentile = 206 m, 95% CI 184–234 m). This diameter could also be roughly estimated from the spatial autocorrelation analysis, given that it corresponds to the distance at which spatial correlogram stops being significantly positive (*x*-intercept = 200–300 m).

Accurately estimated dispersal range is also critical when determining the size of the area targeted by sterile or incompatible mosquito male releases for population elimination (SIT or IIT programs). In Fresno County, California, a high suppression (~ 95%) but not local elimination of *Ae. aegypti* was achieved with very high release numbers of *Wolbachia-*infected males [54]. The inability to achieve complete local elimination was probably the result of immigration of inseminated females from the nearby untreated areas [54]. In this landscape, released males were recaptured in large numbers up to 200 m from the nearest release point, and females were increasingly abundant closer they were to the untreated area (particularly up to 200 m from the treatment edge) [54]. Therefore, expanding the treatment area to account for a buffer of more than 200 m around the core area would likely be needed to permit complete elimination in the core. In Tampines and Yishun, spatial genetic analyses (tail of the dispersal kernel, *x*-intercept) suggest this buffer zone to be ~ 250 m.

The theoretical approximation of the conditions for *Wolbachia*-based mosquito replacement [47] and simulation modeling of this and other mosquito control strategies [10, 55–58] have all been developed assuming isotropic dispersal (invariable based on direction) in a 2-dimensional landscape. The approximation of the dispersal kernel for high-rise uniform landscapes could be achieved by considering the releases of mosquitoes from multiple floors rather than from the ground level only. Clearly, further theoretical and simulation modeling development that incorporates mosquito dispersal that is anisotropic (variable based on direction) in a 3-dimensional habitat is needed to precisely predict the requirements and outcomes of different mosquito control strategies in complex landscapes with a prominent vertical dimension.

## Conclusions

Accurate and precise characterization of dispersal in the field is critical for the optimization of surveillance and control of disease vectors like *Ae. aegypti.* Knowledge of the dispersal kernel parameters enables operational teams to design and implement optimal surveillance, control, and release strategies in a given landscape, facilitating the efficient utilization of resources and maximizing the impacts of interventions. We show that spatial genetic analyses can provide robust estimates of mosquito dispersal patterns. Our newly developed method for the construction of the effective dispersal distance kernel through close-kin analysis enables the most comprehensive estimation of relevant parameters. The indirect inference from the IBD framework that requires less intensive sampling than close-kin analysis can also provide estimates of dispersal kernel spread; however, this approach is sensitive to the inaccurate estimates of effective population size and is uninformative about the probabilities of long-range dispersal that have important implications for control programs. Spatial autocorrelation analysis can complement the IBD analysis to ascertain the spatial extent of the effective dispersal kernel tail through estimation of the genetic patch size. With the decreasing cost of next-generation sequencing, acquisition of spatial genetic data is increasingly accessible. Given the complexities and criticisms of conventional MRR methods, and the central role of dispersal measures in essential vector control programs, we recommend genetic-based dispersal characterization as the more desirable means of parameterization.

## Methods

### Field collections

Adult *Aedes aegypti* females were collected using the sticky traps developed by the Environmental Health Institute, National Environment Agency of Singapore (EHI, NEA), known as the Gravitraps—simple, hay infusion-filled cylindrical traps with a sticky inner surface that preferentially catch gravid females in search of suitable ovipositing sites [25]. The Gravitraps have been deployed in public housing estates island-wide since 2017 as part of the vector surveillance program. For the current study, we chose two public housing estates: Tampines (30 acre sampling area in patch 1, 10 acres in patch 2) and Yishun (46 acre sampling area) that are 14 km apart (Fig. 1a, b). Each unit in the high-rise apartment blocks has open windows and entrance from an open-air corridor suitable for mosquito movement. There are no heating or cooling ducts between apartment blocks and floors that could serve as movement corridors for mosquitoes. The Gravitraps were positioned for vertical sampling in each block: ground level (1st–2nd floors), mid-level (4th–5th floors), and high level (9th floor and above). In both sites, the median number of traps per block per week was 6 (mean = 6.4–6.7). Sticky linings from each Gravitrap were collected by the entomological surveillance team once a week. All adult mosquitoes were identified up to species/genus level using the taxonomic keys. Mosquitoes identified as *Ae. aegypti* were transferred to 2-mL tubes according to their collection date, residential block, and floor and stored in 100% ethanol at 4 °C until further processing.

### DNA extraction, sequencing, and genotyping of individual mosquitoes

Total genomic DNA was extracted from individual mosquitoes using Dneasy Blood and Tissue DNA extraction kit (Qiagen, Hilden, Germany) according to the manufacturer’s instructions. Individual mosquitoes were homogenized manually in 180 μl of ATL buffer using sterile plastic pestles, and the proteinase digestion (with 20 μl of proteinase K) was carried out overnight. The DNA was quantified by using the Qubit High Sensitivity DNA kit (Thermo Fisher Scientific, Waltham, MA, USA), and only the specimens that yielded a DNA concentration of at least 4 ng/μL were included in RADseq libraries. Reduced-genome representation sequence data were generated for each individual using the double-digest RAD sequencing approach by Peterson et al. [26], with the sample processing and library preparation protocol as described in Rašić et al. [27]. ddRAD-seq libraries were sequenced on the Illumina HiSeq4000 platform. The sequencing data were demultiplexed [59] and processed (trimmed to 90 bp and filtered for quality) using the bash script/pipeline from Rašić et al. [27]. The percentages of raw reads passing the quality filtering threshold (phred score ≥ 20) were high for all individuals (96.52–98.29%), suggesting no substantial DNA degradation [60]. The high-quality reads were aligned to the AaegL5 version of the *Ae. aegypti* genome assembly [61] using Bowtie [62]. Unambiguously mapped high-quality reads were converted to bam format and processed in SAMtools [63] to generate sorted bam files that were used to produce genotype likelihood and VCF files using the SAMtools variant calling method as implemented in ANGSD [64]. The final dataset included 107 mosquitoes from Tampines and 108 from Yishun that had < 30% missing data at 83,255 and 69,051 variable sites (SNPs) for the Tampines and Yishun datasets, respectively.

### Inference of familial relationship (kinship estimation)

Relationships between individuals were determined using the recently developed approach by Waples et al. [28] as implemented in the program NGSRelate [29]. This method shows improved accuracy and precision when compared to related approaches, given that (a) it does not require population allele frequency estimates; instead, the framework calculates two-dimensional site-frequency-spectrum for each pair of individuals, and (b) it is applied directly to sequencing data (via genotype likelihoods) rather than the called genotypes, which is particularly suitable for lower-depth sequencing data acquired in RAD-seq experiments [28]. Like most other relatedness inference methods, it can be biased upward or downward for different violations of underlying assumptions (inbreeding, population structure or admixture) [28]. Analogous to the KING-robust method [65], this method is expected to generally be negatively biased for pairs of individuals that have different ancestries, but it is also fairly robust in separating close kin with similar ancestry from unrelated individuals under population structure [30, 31].

For the spatial analyses, we considered close kin as pairs with an inferred category of 1st-, 2nd-, or 3rd-degree relatives. Kinship categories were determined based on the combination of three statistics (R0, R1, and KING-robust kinship), which allows the distinction between the parent-offspring and the full-sibling relationship within the category of 1st-degree relatives [28]. Second-degree relatives include half-siblings, avuncular, and grandparent-grandoffspring pairs that cannot be genetically distinguished, but the grandparent-grandoffspring category is the least likely in our sampling scheme (collection of gravid females, ≥ 80% collected in 1 week and > 93% collected during 2 weeks in each site). Also, we can assume that half-siblings are paternal (i.e., half-sisters share a father, not a mother) given that *Ae. aegypti* females rarely mate more than once [66, 67]. We assume that 3rd-degree relatives are first cousins, given that a category like great-grandparent/great-grandoffspring is unlikely in our sampling scheme.

### Genetic and geographic distance among individuals

We used different individual-based genetic distances among individuals within each area (Yishun or Tampines). The PCA-based genetic distance was estimated by first performing the principal component analysis (PCA) from genotype data in the R package “adegenet” [68] and then creating a distance matrix from the Euclidean distance among the maximal number of PC axes. PCA genetic distance does not assume any particular microevolutionary processes, and it exhibits a linear relationship with Euclidean geographic distance, showing the highest model selection accuracy in landscape genetic studies, especially when dispersal rates are high across the examined area [37]. We also estimated Rousset’s genetic distance *â* [36] and Loiselle’s kinship coefficient [38] in the program SPAGeDI [69].

Pairwise spatial (geographic) distance between mosquitoes was calculated as the shortest straight line (Euclidean) distance in three dimensions, based on the *X*/*Y* (long/lat) and *Z* (height) coordinates of their collection point, here called the Euclidean 3D distance, representing a linear geographic distance in meters (m). Natural logarithm (ln) of this distance was used in the analyses where Rousset’s *â* or Loiselle’s kinship coefficient was applied (see below), given that both of these genetic coefficients exhibit approximate linear relationship with ln-geographic distance [69].

### Estimation of mosquito dispersal characteristics

#### Dispersal kernel estimation from close-kin data

In our sampling scheme, adult females were caught after landing on a lethal ovipositing site (Gravitrap), and we assume that this is a result of their active flight (and not passive, human-assisted movement). We consider pairs of females caught in different traps (non-zero separation distance) that could be genetically assigned to one of the following kinship categories: parent-offspring (*po*), full siblings (*fs*), 2nd-degree relatives (*2nd*) (half siblings, *hs*; avuncular, *av*; grandparent-grandoffspring, *go*), 3rd-degree relatives (*3rd*) (first cousins, *fc*), and non-close kin. Every close-kin category contains information about the number of possible effective dispersal events. For example, a pair of full siblings (*fs*) could have originated from the same breeding site from which each sibling moved into a gravitrap (two events), or they could have originated from different breeding sites (three events, including mother’s skip oviposition). Therefore, the corresponding number of possible dispersal events, for each case, can be calculated as the number of such breeding sites (*n*) plus one (*n* + 1). Based on this, we constructed the sets with elements that represent the number of possible dispersal events for each case as {*n*_min_ + 1,.., *n*_max_ + 1}. For the *fs* category, this set is FS = {2, 3}. In the case of a parent-offspring (*po*) pair, the minimum and maximum number of breeding sites is *n*_min_ = *n*_max_ = 1, giving a set PO = {2}. For the kinship category of 2nd-degree relatives, we have the following subsets: half siblings HS = {2, 3, 4, 5}, avuncular AV = {3, 4}, and grandparent-grandoffspring GO = {2, 3}. We constructed the full set for 2nd-degree relatives as the union of these three subsets (containing unique elements): 2ND = HS ∪ AV ∪ GO = {2, 3, 4, 5}. In the case of 3rd-degree relatives (first cousins *fc*), the minimum number of contributing breeding sites is *n*_min_ = 1 (first cousins and their mothers all originate from the same breeding site), while the maximum is *n*_max_ = 4 (first cousins and their mothers each originate from a unique breeding site). Therefore, for the category of 3rd-degree relatives, we can construct a set as 3RD = FC = {2, 3, 4, 5}.

We then created a set of possible effective dispersal distances for each detected close-kin pair based on their distance and assigned kinship category. This set of distances contains the same number of elements as the corresponding set of possible effective dispersal events (described above), and its values are obtained by dividing the detected spatial distance between a pair (*d*) with the corresponding set element. For example, if a collected pair AB, separated by distance *d*_AB_, falls into the category of 3rd-degree relatives, then a set of possible effective dispersal distances for this pair would be *d*_3rd__,__AB_ = {*d*_AB_/2, *d*_AB_/3, *d*_AB_/4, *d*_AB_/5}. For a full-sibling pair BC separated by spatial distance *d*_BC_, the set of possible effective dispersal distances will be *d*_fs,BC_ = {*d*_BC_/2, *d*_BC_/3}.

By combining the values from all pairwise sets of possible effective dispersal distances into one dataset, we characterized the resulting distribution of possible effective dispersal distances. This dataset was used to generate a probability density function (*pdf*) of effective dispersal distance (i.e., effective dispersal distance kernel) by fitting different functions (exponential, Weibull, log-normal) using the R package “fitdistrplus” [33] that incorporates maximum likelihood estimation and parametric bootstrapping to generate median as well as 2.5 and 97.5 percentiles of each distribution parameter. To determine the “best fitting” of the tested distributions, we assessed the Q-Q plots and computed goodness-of-fit statistics with an approximate Kolmogorov-Smirnov test, Akaike Information Criterion (AIC), and Bayesian Information Criterion (BIC) [33].

To estimate a *pdf* for randomly spaced individuals across the sampled area (null distribution), we simulated 100 datasets where pairs had a randomly assigned kinship category and a distance randomly sampled from the recorded distances between non-close kin. The number of simulated pairs in each kinship category matched the number of observed pairs in the empirical dataset. We then applied the analytical procedure described above on all simulated datasets and compared the simulated (random) and empirical distributions in the R package “sm” [70] using the permutation test of equality of two distribution densities.

#### Isolation-by-distance analysis (IBD) and estimation of the dispersal kernel spread

IBD analysis can be used even when few or no close kin are captured. In fact, highly related individuals should be removed from the IBD analysis in order for it to reflect the long-term population processes [71], and we created a subsample for each area by removing individuals identified as close kin, leaving 63 and 85 individuals in the Tampines and Yishun subsample, respectively. The significance of IBD was tested separately in Tampines and Yishun using Mantel’s correlation test with 1000 permutations, as implemented in the R package “ecodist” [72].

IBD is best illustrated by the regression of pairwise genetic distances onto geographic distances among individuals [34]. The slope of this linear regression and the effective density can be used to estimate the standard deviation of the dispersal kernel (σ) that is also known as the dispersal kernel spread [50]. The dispersal kernel spread can be calculated as σ = √(1/4π*Db*), where *b* is the slope of the linear regression and *D* is the effective density of reproducing individuals.

The slope of the linear relationship was estimated using the lm() function in R (R Core Team) for three different sets of genetic and geographic matrices. The first set included a matrix of PCA-based genetic distances against the matrix of linear geographic distances. A matrix of Rousset’s *â* or Loiselle’s kinship coefficient was tested against the matrix of ln-transformed geographic distances, given that both genetic estimators are expected to vary approximately linearly with the natural logarithm of the distance [37].

The effective density *D* is defined as *N*_*e*_/study area, where *N*_*e*_ is the effective population size. We estimated *N*_*e*_ using two genetic methods based on a single sample. The first method is *N*_*e*_ estimation by Waples and Waples [39], based on the “parentage analysis without parents” (method 1, PWoP) that uses the frequency of full- and half-siblings in a population sample to reconstruct the number of parents that contributed to such a sample*.* The median and the 95% confidence interval were calculated using 100 resamples with a random replacement of one individual. The second method represents *N*_*e*_ estimation by Waples and England [40] that is based on the linkage disequilibrium data (method 2, LDN_e_), with the 95% confidence interval calculated using the jackknifing procedure over loci implemented in the program *N*_*e*_ estimator v.2.1 [73]. Finally, effective density was estimated using the entomological survey data from the Gravitrap sentinel trap system across the study areas for the period from January 2018 through May 2018 (method 3, Gravitrap). During those months, the surveillance system contained a minimum of 1357 traps distributed over a 45 acre area in Tampines and a minimum of 1048 traps over a 65 acre area in Yishun. An average of 441 adult females (range 391–760) were caught per month in Tampines, and 280 (range 202–404) in Yishun (Additional file 1: Table S7). The average number of breeding females per square meter was multiplied by 2 to give the effective number of breeding individuals per unit area, as we assume 1:1 sex ratio in an *Ae. aegypti* population [74]. For Tampines, we considered patch 1 (larger sampling area) as a more representative population sample for the calculations of local *N*_*e*_ and density than the smaller patch 2 (Fig. 1a).

#### Spatial autocorrelation analysis

To compute the correlogram curve for each sampling site, we used PCA genetic distance among all genotyped mosquitoes in a site, and the spatial grouping within distance classes that were incrementally increased by 50 m. The analysis was done in GenAlEx v.6.501 [75]. The autocorrelation coefficient under the null hypothesis of no spatial structure was generated by the permutation procedure that shuffles all individuals among the geographic locations within a site 1000 times and generates 95% CI with the 25th and 975th ranked permutated values. 95% CI for the observed autocorrelation coefficient for each distance class was obtained from 1000 random draws of individuals with replacement.

## Supplementary information

**Additional file 1: Table S1.** Tampines: the number of female *Aedes aegypti* caught per week per building. **Table S2.** Tampines: the number of recovered traps per building. **Table S3.** Yishun: the number of female *Aedes aegypti* caught per building. **Table S4.** Yishun: the number of recovered traps per building. **Table S5.** IBD-based estimates of the dispersal kernel spread (σ) in *Aedes aegypti* from Singapore. **Table S6.** Mantel and partial Mantel tests for non-close-kin data in Tampines and Yishun. **Table S7.** Entomological data used for the estimation of effective population density in *Aedes aegypti* (method 3).

**Additional file 2: Fig. S1.** Spatial network of close kin in Tampines (A) and Yishun (B). **Fig. S2.** Frequency histogram of separation distance for each kinship category. **Fig. S3.** Distribution fitting (dispersal kernel parametrization) analysis.

## Data Availability

The datasets generated and analyzed during the current study are available in the NCBI Sequence Read Archive (SRA) repository under the accession number PRJNA639373, https://www.ncbi.nlm.nih.gov/bioproject/PRJNA639373 [59], and are included in this published article and its Additional files.
