# Supplementary material for: Using spatial genetics to quantify mosquito dispersal for control programs
Source: BMC Biol. 2020 Aug 20;18:104. doi: 10.1186/s12915-020-00841-0 (PMC7439557; doi:10.1186/s12915-020-00841-0)
Supplement: Supplementary file 1 — Additional file 1: Table S1. Tampines: the number of female Aedes aegypti caught per week per building. Table S2. Tampines: the number of recovered traps per building. Table S3. Yishun: the number of female Aedes aegypti caught per building. Table S4. Yishun: the number of recovered traps per building. Table S5. IBD-based estimates of the dispersal kernel spread (σ) in Aedes aegypti from Singapore. Table S6. Mantel and partial Mantel tests for non-close-kin data in Tampines and Yishun. Table S7. Entomological data used for the estimation of effective population density in Aedes aegypti (method 3). [file 12915_2020_841_MOESM1_ESM.docx]

Additional file 1. TableS1. Tampines: the number of female *Aedes aegypti* caught per week per building

| **Building** | **Site** | **Epidemiological week 2018** | | | | | | | | | | | | | | | | | | | | | |
| --- | --- | --- | --- | --- | --- | --- | --- | --- | --- | --- | --- | --- | --- | --- | --- | --- | --- | --- | --- | --- | --- | --- | --- |
|  |  | **1** | **2** | **3** | **4** | **5** | **6** | **7** | **8** | **9** | **10** | **11** | **12** | **13** | **14** | **15** | **16** | **17** | **18** | **19** | **20** | **21** | **22** |
| 803 | Tampines (patch 1) | 1 | 0 | 1 | 1 | 1 | 2 | 4 | 2 | 0 | 0 | 0 | 1 | 0 | 2 | 1 | 0 | 4 | 1 | 2 | 4 | 4 | 0 |
| 804 | Tampines (patch 1) | 1 | 4 | 0 | 5 | 6 | 4 | 3 | 3 | 0 | 0 | 0 | 0 | 0 | 2 | 2 | 2 | 6 | 0 | 4 | 0 | 1 | 5 |
| 805 | Tampines (patch 1) | 8 | 0 | 7 | 7 | 5 | 4 | 2 | 3 | 2 | 5 | 0 | 4 | 1 | 0 | 1 | 0 | 15 | 3 | 9 | 4 | 3 | 1 |
| 806 | Tampines (patch 1) | 2 | 3 | 0 | 6 | 2 | 2 | 2 | 2 | 1 | 0 | 1 | 2 | 0 | 2 | 4 | 4 | 12 | 4 | 8 | 7 | 0 | 2 |
| 807 | Tampines (patch 1) | 4 | 5 | 3 | 5 | 2 | 6 | 2 | 5 | 2 | 2 | 3 | 1 | 3 | 3 | 14 | 11 | 14 | 3 | 3 | 2 | 5 | 1 |
| 809 | Tampines (patch 1) | 4 | 1 | 5 | 8 | 7 | 1 | 8 | 3 | 3 | 4 | 10 | 7 | 14 | 12 | 10 | 26 | 5 | 5 | 1 | 2 | 2 | 6 |
| 810 | Tampines (patch 1) | 3 | 6 | 7 | 18 | 7 | 5 | 6 | 6 | 8 | 2 | 10 | 13 | 25 | 25 | 19 | 34 | 16 | 5 | 26 | 2 | 5 | 1 |
| 811 | Tampines (patch 1) | 7 | 3 | 4 | 2 | 4 | 4 | 4 | 8 | 3 | 1 | 0 | 2 | 1 | 1 | 1 | 2 | 8 | 6 | 8 | 2 | 3 | 4 |
| 812 | Tampines (patch 1) | 13 | 2 | 9 | 4 | 2 | 5 | 12 | 4 | 1 | 1 | 1 | 2 | 1 | 5 | 1 | 1 | 3 | 3 | 15 | 2 | 1 | 7 |
| 813 | Tampines (patch 1) | 10 | 8 | 2 | 10 | 6 | 5 | 5 | 8 | 10 | 2 | 0 | 8 | 13 | 16 | 10 | 21 | 20 | 8 | 15 | 2 | 11 | 5 |
| 814 | Tampines (patch 1) | 0 | 2 | 2 | 1 | 1 | 0 | 2 | 2 | 2 | 5 | 1 | 4 | 11 | 7 | 12 | 16 | 26 | 13 | 44 | 1 | 2 | 5 |
| 815 | Tampines (patch 1) | 7 | 7 | 1 | 6 | 9 | 10 | 19 | 18 | 5 | 4 | 0 | 4 | 6 | 2 | 5 | 1 | 11 | 5 | 9 | 2 | 1 | 1 |
| 816 | Tampines (patch 1) | 2 | 0 | 1 | 4 | 5 | 0 | 1 | 1 | 1 | 2 | 1 | 4 | 2 | 7 | 0 | 5 | 12 | 7 | 18 | 13 | 1 | 9 |
| 818 | Tampines (patch 1) | 0 | 0 | 0 | 0 | 3 | 2 | 4 | 0 | 3 | 0 | 0 | 0 | 0 | 2 | 1 | 2 | 4 | 1 | 6 | 6 | 0 | 9 |
| 898 | Tampines (patch 1) | 2 | 0 | 1 | 0 | 1 | 3 | 1 | 0 | 0 | 0 | 0 | 2 | 3 | 6 | 3 | 8 | 10 | 3 | 7 | 7 | 6 | 4 |
| 899 | Tampines (patch 1) | 0 | 0 | 0 | 5 | 0 | 7 | 0 | 3 | 0 | 2 | 2 | 6 | 2 | 1 | 5 | 1 | 8 | 3 | 3 | 6 | 4 | 2 |
| 890C | Tampines (patch 1) | 1 | 0 | 0 | 1 | 1 | 0 | 1 | 0 | 0 | 0 | 0 | 0 | 0 | 0 | 2 | 1 | 0 | 1 | 2 | 2 | 3 | 1 |
| 897A | Tampines (patch 1) | 0 | 1 | 0 | 0 | 2 | 0 | 2 | 0 | 0 | 0 | 0 | 1 | 1 | 1 | 2 | 1 | 4 | 4 | 7 | 7 | 7 | 4 |
| 898A | Tampines (patch 1) | 1 | 4 | 0 | 4 | 2 | 0 | 2 | 1 | 2 | 4 | 2 | 5 | 4 | 6 | 4 | 6 | 4 | 2 | 3 | 7 | 2 | 3 |
| 899A | Tampines (patch 1) | 1 | 0 | 0 | 1 | 4 | 3 | 2 | 1 | 0 | 0 | 5 | 5 | 2 | 2 | 2 | 0 | 2 | 1 | 2 | 1 | 5 | 3 |

TableS2. Tampines: the number of recovered traps per building.

| **Building** | **Site** | **Epidemiological week 2018** | | | | | | | | | | | | | | | | | | | | | |
| --- | --- | --- | --- | --- | --- | --- | --- | --- | --- | --- | --- | --- | --- | --- | --- | --- | --- | --- | --- | --- | --- | --- | --- |
|  |  | **1** | **2** | **3** | **4** | **5** | **6** | **7** | **8** | **9** | **10** | **11** | **12** | **13** | **14** | **15** | **16** | **17** | **18** | **19** | **20** | **21** | **22** |
| 803 | Tampines (patch 1) | 6 | 6 | 6 | 6 | 6 | 6 | 6 | 6 | 6 | 6 | 6 | 6 | 6 | 6 | 6 | 6 | 6 | 6 | 6 | 6 | 6 | 6 |
| 804 | Tampines (patch 1) | 6 | 6 | 6 | 6 | 6 | 6 | 6 | 6 | 6 | 6 | 6 | 6 | 6 | 6 | 6 | 6 | 6 | 6 | 6 | 6 | 6 | 6 |
| 805 | Tampines (patch 1) | 6 | 6 | 6 | 6 | 6 | 6 | 6 | 6 | 6 | 6 | 6 | 6 | 6 | 6 | 6 | 6 | 6 | 6 | 6 | 6 | 6 | 6 |
| 806 | Tampines (patch 1) | 6 | 6 | 6 | 6 | 6 | 6 | 6 | 6 | 6 | 6 | 6 | 6 | 6 | 6 | 6 | 6 | 6 | 6 | 6 | 6 | 6 | 6 |
| 807 | Tampines (patch 1) | 6 | 6 | 6 | 6 | 6 | 6 | 6 | 6 | 6 | 6 | 6 | 6 | 6 | 6 | 6 | 6 | 6 | 6 | 6 | 6 | 6 | 6 |
| 809 | Tampines (patch 1) | 6 | 6 | 6 | 6 | 6 | 6 | 6 | 6 | 6 | 6 | 6 | 6 | 6 | 6 | 6 | 6 | 6 | 6 | 6 | 6 | 6 | 6 |
| 810 | Tampines (patch 1) | 6 | 6 | 6 | 6 | 6 | 6 | 6 | 6 | 6 | 6 | 6 | 6 | 6 | 6 | 6 | 6 | 6 | 6 | 6 | 6 | 6 | 6 |
| 811 | Tampines (patch 1) | 6 | 6 | 6 | 6 | 6 | 6 | 6 | 6 | 6 | 6 | 6 | 6 | 6 | 6 | 6 | 6 | 6 | 6 | 6 | 6 | 6 | 6 |
| 812 | Tampines (patch 1) | 6 | 6 | 6 | 6 | 6 | 6 | 6 | 6 | 6 | 6 | 6 | 6 | 6 | 6 | 6 | 6 | 6 | 6 | 6 | 6 | 6 | 6 |
| 813 | Tampines (patch 1) | 6 | 6 | 6 | 6 | 6 | 6 | 6 | 6 | 6 | 6 | 6 | 6 | 6 | 6 | 6 | 6 | 6 | 6 | 6 | 6 | 6 | 6 |
| 814 | Tampines (patch 1) | 6 | 6 | 6 | 6 | 6 | 6 | 6 | 6 | 6 | 6 | 6 | 6 | 6 | 6 | 6 | 6 | 6 | 6 | 6 | 6 | 6 | 6 |
| 815 | Tampines (patch 1) | 8 | 8 | 8 | 8 | 8 | 8 | 8 | 8 | 8 | 8 | 8 | 8 | 8 | 8 | 8 | 8 | 8 | 8 | 8 | 8 | 8 | 8 |
| 816 | Tampines (patch 1) | 8 | 8 | 8 | 8 | 8 | 8 | 8 | 8 | 8 | 8 | 8 | 8 | 8 | 8 | 8 | 8 | 8 | 8 | 8 | 8 | 8 | 8 |
| 818 | Tampines (patch 1) | 6 | 6 | 6 | 6 | 6 | 6 | 6 | 6 | 6 | 6 | 6 | 6 | 6 | 6 | 6 | 6 | 6 | 6 | 6 | 6 | 6 | 6 |
| 898 | Tampines (patch 1) | 6 | 6 | 6 | 6 | 6 | 6 | 6 | 6 | 6 | 6 | 6 | 6 | 6 | 6 | 6 | 6 | 6 | 6 | 6 | 6 | 6 | 6 |
| 899 | Tampines (patch 1) | 6 | 6 | 6 | 6 | 6 | 6 | 6 | 6 | 6 | 6 | 6 | 6 | 6 | 6 | 6 | 6 | 6 | 6 | 6 | 6 | 6 | 6 |
| 890C | Tampines (patch 1) | 8 | 8 | 8 | 8 | 8 | 8 | 8 | 8 | 8 | 8 | 8 | 8 | 8 | 8 | 8 | 8 | 8 | 8 | 8 | 8 | 8 | 8 |
| 897A | Tampines (patch 1) | 6 | 6 | 6 | 6 | 6 | 6 | 6 | 6 | 6 | 6 | 6 | 6 | 6 | 6 | 6 | 6 | 6 | 6 | 6 | 6 | 6 | 6 |
| 898A | Tampines (patch 1) | 8 | 8 | 8 | 8 | 8 | 8 | 8 | 8 | 8 | 8 | 8 | 8 | 8 | 8 | 8 | 8 | 8 | 8 | 8 | 8 | 8 | 8 |
| 899A | Tampines (patch 1) | 6 | 6 | 6 | 6 | 6 | 6 | 6 | 6 | 6 | 6 | 6 | 6 | 6 | 6 | 6 | 6 | 6 | 6 | 6 | 6 | 6 | 6 |

TableS3. Yishun: the number of female *Aedes aegypti* caught per building.

| **Building** | **Site** | **Epidemiological week 2018** | | | | | | | | | | | | | | | | | | | | | |
| --- | --- | --- | --- | --- | --- | --- | --- | --- | --- | --- | --- | --- | --- | --- | --- | --- | --- | --- | --- | --- | --- | --- | --- |
|  |  | **1** | **2** | **3** | **4** | **5** | **6** | **7** | **8** | **9** | **10** | **11** | **12** | **13** | **14** | **15** | **16** | **17** | **18** | **19** | **20** | **21** | **22** |
| 201 | Yishun | 1 | 0 | 8 | 12 | 9 | 9 | 5 | 2 | 4 | 3 | 2 | 0 | 3 | 0 | 0 | 3 | 3 | 3 | 2 | 7 | 1 | 2 |
| 202 | Yishun | 0 | 3 | 5 | 8 | 4 | 2 | 1 | 1 | 2 | 4 | 4 | 1 | 3 | 0 | 0 | 1 | 4 | 4 | 0 | 3 | 1 | 0 |
| 203 | Yishun | 3 | 2 | 6 | 5 | 4 | 2 | 2 | 2 | 2 | 1 | 0 | 1 | 2 | 2 | 1 | 3 | 5 | 1 | 2 | 4 | 1 | 2 |
| 205 | Yishun | 9 | 8 | 4 | 3 | 5 | 11 | 4 | 3 | 2 | 0 | 2 | 0 | 6 | 4 | 5 | 3 | 4 | 3 | 2 | 3 | 1 | 1 |
| 208 | Yishun | 5 | 2 | 3 | 1 | 6 | 6 | 2 | 0 | 2 | 0 | 5 | 3 | 3 | 1 | 1 | 3 | 1 | 1 | 2 | 0 | 3 | 0 |
| 209 | Yishun | 1 | 4 | 9 | 0 | 2 | 3 | 1 | 4 | 3 | 0 | 2 | 2 | 0 | 1 | 0 | 2 | 3 | 1 | 0 | 1 | 1 | 0 |
| 210 | Yishun | 2 | 1 | 4 | 0 | 4 | 6 | 0 | 3 | 1 | 0 | 3 | 0 | 2 | 1 | 3 | 2 | 6 | 0 | 0 | 2 | 2 | 0 |
| 211 | Yishun | 1 | 3 | 9 | 18 | 9 | 4 | 0 | 0 | 3 | 0 | 2 | 0 | 2 | 1 | 1 | 1 | 6 | 2 | 1 | 3 | 3 | 2 |
| 212 | Yishun | 1 | 8 | 1 | 5 | 4 | 1 | 0 | 1 | 2 | 2 | 1 | 2 | 2 | 3 | 1 | 0 | 2 | 0 | 0 | 2 | 0 | 0 |
| 213 | Yishun | 1 | 3 | 3 | 0 | 3 | 1 | 1 | 1 | 0 | 1 | 1 | 0 | 0 | 5 | 0 | 1 | 3 | 4 | 7 | 1 | 1 | 0 |
| 214 | Yishun | 0 | 5 | 2 | 4 | 2 | 3 | 1 | 5 | 2 | 2 | 2 | 7 | 2 | 3 | 4 | 4 | 4 | 4 | 3 | 4 | 5 | 1 |
| 215 | Yishun | 0 | 1 | 1 | 1 | 2 | 3 | 0 | 2 | 2 | 2 | 5 | 0 | 1 | 2 | 2 | 3 | 9 | 3 | 3 | 4 | 0 | 3 |
| 216 | Yishun | 2 | 0 | 0 | 0 | 2 | 2 | 1 | 2 | 2 | 1 | 3 | 1 | 1 | 2 | 1 | 0 | 2 | 2 | 5 | 2 | 0 | 0 |
| 217 | Yishun | 1 | 0 | 2 | 2 | 3 | 0 | 0 | 3 | 2 | 0 | 1 | 0 | 1 | 2 | 0 | 3 | 5 | 4 | 1 | 2 | 0 | 5 |
| 218 | Yishun | 4 | 1 | 2 | 2 | 0 | 2 | 2 | 0 | 0 | 0 | 1 | 0 | 1 | 0 | 0 | 3 | 2 | 1 | 1 | 1 | 0 | 2 |
| 219 | Yishun | 3 | 2 | 4 | 2 | 8 | 1 | 0 | 0 | 1 | 0 | 0 | 0 | 0 | 1 | 1 | 1 | 0 | 0 | 0 | 0 | 0 | 3 |
| 220 | Yishun | 3 | 2 | 0 | 1 | 6 | 0 | 2 | 4 | 1 | 2 | 2 | 3 | 1 | 2 | 1 | 2 | 1 | 1 | 3 | 1 | 0 | 0 |
| 221 | Yishun | 0 | 2 | 3 | 0 | 0 | 1 | 0 | 5 | 2 | 1 | 1 | 1 | 1 | 1 | 0 | 0 | 3 | 1 | 0 | 0 | 0 | 0 |
| 222 | Yishun | 2 | 3 | 0 | 2 | 5 | 3 | 2 | 2 | 1 | 0 | 1 | 1 | 2 | 5 | 5 | 4 | 6 | 3 | 3 | 4 | 0 | 2 |
| 223 | Yishun | 2 | 4 | 1 | 0 | 4 | 2 | 1 | 2 | 1 | 1 | 1 | 5 | 3 | 3 | 2 | 0 | 1 | 5 | 6 | 6 | 1 | 2 |
| 224 | Yishun | 1 | 0 | 2 | 0 | 0 | 1 | 0 | 2 | 2 | 3 | 1 | 0 | 0 | 0 | 0 | 6 | 0 | 2 | 8 | 2 | 3 | 3 |
| 225 | Yishun | 0 | 1 | 0 | 2 | 1 | 1 | 0 | 1 | 0 | 0 | 2 | 0 | 0 | 1 | 2 | 4 | 4 | 3 | 4 | 3 | 0 | 2 |
| 226 | Yishun | 0 | 0 | 0 | 1 | 1 | 1 | 1 | 0 | 0 | 0 | 0 | 0 | 0 | 0 | 0 | 1 | 1 | 2 | 0 | 0 | 1 | 0 |
| 227 | Yishun | 1 | 1 | 1 | 0 | 1 | 0 | 0 | 0 | 0 | 2 | 0 | 0 | 0 | 0 | 1 | 2 | 2 | 2 | 4 | 3 | 3 | 1 |
| 230 | Yishun | 0 | 0 | 0 | 2 | 2 | 1 | 0 | 1 | 1 | 0 | 1 | 0 | 0 | 1 | 4 | 1 | 6 | 2 | 0 | 0 | 2 | 0 |
| 231 | Yishun | 1 | 0 | 1 | 0 | 4 | 2 | 1 | 2 | 1 | 2 | 2 | 0 | 0 | 2 | 1 | 0 | 5 | 4 | 2 | 0 | 2 | 2 |
| 232 | Yishun | 4 | 4 | 2 | 0 | 1 | 0 | 1 | 1 | 1 | 2 | 1 | 1 | 0 | 0 | 0 | 0 | 3 | 0 | 1 | 0 | 1 | 0 |
| 233 | Yishun | 0 | 0 | 0 | 0 | 0 | 0 | 0 | 0 | 0 | 0 | 0 | 0 | 0 | 0 | 1 | 0 | 0 | 0 | 0 | 0 | 0 | 1 |
| 234 | Yishun | 0 | 0 | 0 | 0 | 0 | 1 | 1 | 4 | 1 | 0 | 0 | 1 | 0 | 1 | 2 | 3 | 1 | 1 | 3 | 0 | 1 | 1 |
| 235 | Yishun | 0 | 0 | 2 | 0 | 3 | 1 | 1 | 1 | 0 | 0 | 1 | 1 | 0 | 0 | 0 | 0 | 3 | 3 | 1 | 1 | 1 | 0 |
| 121 | Yishun | 2 | 0 | 0 | 0 | 1 | 0 | 0 | 0 | 1 | 0 | 0 | 0 | 0 | 0 | 0 | 0 | 0 | 2 | 1 | 3 | 1 | 0 |
| 125 | Yishun | 0 | 1 | 0 | 1 | 2 | 0 | 1 | 0 | 1 | 0 | 0 | 0 | 1 | 1 | 0 | 1 | 2 | 0 | 1 | 0 | 0 | 0 |
| 127 | Yishun | 0 | 1 | 0 | 1 | 1 | 1 | 1 | 3 | 1 | 0 | 0 | 1 | 0 | 1 | 0 | 2 | 1 | 0 | 4 | 2 | 1 | 0 |
| 130 | Yishun | 1 | 0 | 1 | 2 | 2 | 1 | 0 | 0 | 0 | 1 | 0 | 0 | 0 | 0 | 0 | 0 | 1 | 0 | 3 | 1 | 2 | 0 |
| 152 | Yishun | 3 | 3 | 4 | 4 | 3 | 3 | 0 | 0 | 0 | 0 | 0 | 0 | 1 | 1 | 2 | 2 | 1 | 1 | 1 | 1 | 0 | 0 |
| 154 | Yishun | 1 | 1 | 0 | 0 | 1 | 1 | 2 | 2 | 3 | 3 | 1 | 1 | 1 | 1 | 1 | 1 | 1 | 1 | 2 | 2 | 2 | 2 |
| 155 | Yishun | 1 | 1 | 1 | 1 | 1 | 0 | 0 | 0 | 0 | 0 | 0 | 1 | 1 | 0 | 0 | 1 | 1 | 0 | 0 | 1 | 1 | 1 |
| 156 | Yishun | 1 | 1 | 1 | 1 | 1 | 1 | 0 | 0 | 1 | 1 | 0 | 0 | 2 | 2 | 1 | 1 | 1 | 1 | 1 | 1 | 1 | 1 |
| 157 | Yishun | 2 | 2 | 2 | 1 | 1 | 1 | 1 | 1 | 1 | 0 | 0 | 1 | 1 | 0 | 0 | 1 | 1 | 1 | 1 | 2 | 2 | 1 |
| 158 | Yishun | 1 | 1 | 1 | 1 | 2 | 2 | 2 | 2 | 1 | 1 | 3 | 3 | 2 | 2 | 1 | 1 | 2 | 2 | 3 | 3 | 0 | 0 |
| 161 | Yishun | 1 | 1 | 1 | 5 | 5 | 2 | 2 | 3 | 3 | 1 | 1 | 3 | 3 | 0 | 0 | 1 | 1 | 0 | 0 | 0 | 0 | 0 |
| 162 | Yishun | 0 | 0 | 2 | 2 | 1 | 1 | 3 | 3 | 3 | 3 | 3 | 3 | 1 | 1 | 2 | 2 | 3 | 3 | 2 | 2 | 1 | 1 |
| 296 | Yishun | 1 | 1 | 3 | 3 | 2 | 2 | 2 | 2 | 2 | 2 | 1 | 1 | 1 | 1 | 1 | 1 | 3 | 3 | 0 | 0 | 2 | 2 |

continued Table S3.

TableS4. Yishun: the number of recovered traps per building.

| **Building** | **Site** | **Epidemiological week 2018** | | | | | | | | | | | | | | | | | | | | | |
| --- | --- | --- | --- | --- | --- | --- | --- | --- | --- | --- | --- | --- | --- | --- | --- | --- | --- | --- | --- | --- | --- | --- | --- |
|  |  | **1** | **2** | **3** | **4** | **5** | **6** | **7** | **8** | **9** | **10** | **11** | **12** | **13** | **14** | **15** | **16** | **17** | **18** | **19** | **20** | **21** | **22** |
| 201 | Yishun | 8 | 8 | 8 | 8 | 8 | 8 | 8 | 8 | 8 | 8 | 8 | 8 | 8 | 8 | 8 | 8 | 8 | 8 | 8 | 8 | 8 | 8 |
| 202 | Yishun | 9 | 9 | 9 | 9 | 9 | 9 | 9 | 9 | 9 | 9 | 9 | 9 | 9 | 9 | 9 | 9 | 9 | 9 | 9 | 9 | 9 | 9 |
| 203 | Yishun | 9 | 9 | 9 | 9 | 9 | 9 | 9 | 9 | 9 | 9 | 9 | 9 | 9 | 9 | 9 | 9 | 9 | 9 | 9 | 9 | 9 | 9 |
| 205 | Yishun | 9 | 9 | 9 | 9 | 9 | 9 | 9 | 9 | 9 | 9 | 9 | 9 | 9 | 9 | 9 | 9 | 9 | 9 | 9 | 9 | 9 | 9 |
| 208 | Yishun | 6 | 6 | 6 | 6 | 6 | 6 | 6 | 6 | 6 | 6 | 6 | 6 | 6 | 6 | 6 | 6 | 6 | 6 | 6 | 6 | 6 | 6 |
| 209 | Yishun | 6 | 6 | 6 | 6 | 6 | 6 | 6 | 6 | 6 | 6 | 6 | 6 | 6 | 6 | 6 | 6 | 6 | 6 | 6 | 6 | 6 | 6 |
| 210 | Yishun | 9 | 9 | 9 | 9 | 9 | 9 | 9 | 9 | 9 | 9 | 9 | 9 | 9 | 9 | 9 | 9 | 9 | 9 | 9 | 9 | 9 | 9 |
| 211 | Yishun | 9 | 9 | 9 | 9 | 9 | 9 | 9 | 9 | 9 | 9 | 9 | 9 | 9 | 9 | 9 | 9 | 9 | 9 | 9 | 9 | 9 | 9 |
| 212 | Yishun | 9 | 9 | 9 | 9 | 9 | 9 | 9 | 9 | 9 | 9 | 9 | 9 | 9 | 9 | 9 | 9 | 9 | 9 | 9 | 9 | 9 | 9 |
| 213 | Yishun | 9 | 9 | 9 | 9 | 9 | 9 | 9 | 9 | 9 | 9 | 9 | 9 | 9 | 9 | 9 | 9 | 9 | 9 | 9 | 9 | 9 | 9 |
| 214 | Yishun | 9 | 9 | 9 | 9 | 9 | 9 | 9 | 9 | 9 | 9 | 9 | 9 | 9 | 9 | 9 | 9 | 9 | 9 | 9 | 9 | 9 | 9 |
| 215 | Yishun | 6 | 6 | 6 | 6 | 6 | 6 | 6 | 6 | 6 | 6 | 6 | 6 | 6 | 6 | 6 | 6 | 6 | 6 | 6 | 6 | 6 | 6 |
| 216 | Yishun | 9 | 9 | 9 | 9 | 9 | 9 | 9 | 9 | 9 | 9 | 9 | 9 | 9 | 9 | 9 | 9 | 9 | 9 | 9 | 9 | 9 | 9 |
| 217 | Yishun | 9 | 9 | 9 | 9 | 9 | 9 | 9 | 9 | 9 | 9 | 9 | 9 | 9 | 9 | 9 | 9 | 9 | 9 | 9 | 9 | 9 | 9 |
| 218 | Yishun | 6 | 6 | 6 | 6 | 6 | 6 | 6 | 6 | 6 | 6 | 6 | 6 | 6 | 6 | 6 | 6 | 6 | 6 | 6 | 6 | 6 | 6 |
| 219 | Yishun | 6 | 6 | 6 | 6 | 6 | 6 | 6 | 6 | 6 | 6 | 6 | 6 | 6 | 6 | 6 | 6 | 6 | 6 | 6 | 6 | 6 | 6 |
| 220 | Yishun | 6 | 6 | 6 | 6 | 6 | 6 | 6 | 6 | 6 | 6 | 6 | 6 | 6 | 6 | 6 | 6 | 6 | 6 | 6 | 6 | 6 | 6 |
| 221 | Yishun | 6 | 6 | 6 | 6 | 6 | 6 | 6 | 6 | 6 | 6 | 6 | 6 | 6 | 6 | 6 | 6 | 6 | 6 | 6 | 6 | 6 | 6 |
| 222 | Yishun | 9 | 9 | 9 | 9 | 9 | 9 | 9 | 9 | 9 | 9 | 9 | 9 | 9 | 9 | 9 | 9 | 9 | 9 | 9 | 9 | 9 | 9 |
| 223 | Yishun | 9 | 9 | 9 | 9 | 9 | 9 | 9 | 9 | 9 | 9 | 9 | 9 | 9 | 9 | 9 | 9 | 9 | 9 | 9 | 9 | 9 | 9 |
| 224 | Yishun | 9 | 9 | 9 | 9 | 9 | 9 | 9 | 9 | 9 | 9 | 9 | 9 | 9 | 9 | 9 | 9 | 9 | 9 | 9 | 9 | 9 | 9 |
| 225 | Yishun | 9 | 9 | 9 | 9 | 9 | 9 | 9 | 9 | 9 | 9 | 9 | 9 | 9 | 9 | 9 | 9 | 9 | 9 | 9 | 9 | 9 | 9 |
| 226 | Yishun | 4 | 4 | 4 | 4 | 4 | 4 | 4 | 4 | 4 | 4 | 4 | 4 | 4 | 4 | 4 | 4 | 4 | 4 | 4 | 4 | 4 | 4 |
| 227 | Yishun | 6 | 6 | 6 | 6 | 6 | 6 | 6 | 6 | 6 | 6 | 6 | 6 | 6 | 6 | 6 | 6 | 6 | 6 | 6 | 6 | 6 | 6 |
| 230 | Yishun | 2 | 2 | 2 | 2 | 2 | 2 | 2 | 2 | 2 | 2 | 2 | 2 | 2 | 2 | 2 | 2 | 2 | 2 | 2 | 2 | 2 | 2 |
| 231 | Yishun | 6 | 6 | 6 | 6 | 6 | 6 | 6 | 6 | 6 | 6 | 6 | 6 | 6 | 6 | 6 | 6 | 6 | 6 | 6 | 6 | 6 | 6 |
| 232 | Yishun | 6 | 6 | 6 | 6 | 6 | 6 | 6 | 6 | 6 | 6 | 6 | 6 | 6 | 6 | 6 | 6 | 6 | 6 | 6 | 6 | 6 | 6 |
| 233 | Yishun | 2 | 2 | 2 | 2 | 2 | 2 | 2 | 2 | 2 | 2 | 2 | 2 | 2 | 2 | 2 | 2 | 2 | 2 | 2 | 2 | 2 | 2 |
| 234 | Yishun | 6 | 6 | 6 | 6 | 6 | 6 | 6 | 6 | 6 | 6 | 6 | 6 | 6 | 6 | 6 | 6 | 6 | 6 | 6 | 6 | 6 | 6 |
| 235 | Yishun | 6 | 6 | 6 | 6 | 6 | 6 | 6 | 6 | 6 | 6 | 6 | 6 | 6 | 6 | 6 | 6 | 6 | 6 | 6 | 6 | 6 | 6 |
| 121 | Yishun | 8 | 9 | 5 | 8 | 8 | 9 | 9 | 9 | 8 | 9 | 9 | 9 | 7 | 8 | 9 | 9 | 9 | 9 | 8 | 9 | 9 | 9 |
| 125 | Yishun | 6 | 6 | 5 | 6 | 6 | 6 | 6 | 6 | 6 | 6 | 6 | 6 | 6 | 6 | 6 | 6 | 6 | 6 | 6 | 6 | 6 | 6 |
| 127 | Yishun | 6 | 6 | 6 | 6 | 6 | 6 | 6 | 6 | 5 | 6 | 6 | 5 | 5 | 5 | 6 | 6 | 6 | 6 | 6 | 6 | 6 | 6 |
| 130 | Yishun | 9 | 9 | 8 | 9 | 9 | 9 | 9 | 9 | 9 | 9 | 9 | 9 | 8 | 7 | 6 | 8 | 9 | 9 | 9 | 9 | 9 | 9 |
| 152 | Yishun | 4 | 4 | 4 | 4 | 4 | 4 | 4 | 4 | 4 | 4 | 0 | 0 | 4 | 4 | 4 | 4 | 4 | 4 | 4 | 4 | 3 | 3 |
| 154 | Yishun | 6 | 6 | 0 | 0 | 5 | 5 | 6 | 6 | 6 | 6 | 6 | 6 | 6 | 6 | 6 | 6 | 6 | 6 | 6 | 6 | 6 | 6 |
| 155 | Yishun | 6 | 6 | 6 | 6 | 6 | 6 | 6 | 6 | 6 | 6 | 6 | 6 | 6 | 6 | 6 | 5 | 5 | 5 | 5 | 6 | 6 | 6 |
| 156 | Yishun | 2 | 2 | 2 | 2 | 2 | 2 | 2 | 2 | 2 | 2 | 1 | 1 | 2 | 2 | 2 | 2 | 2 | 2 | 1 | 1 | 2 | 2 |
| 157 | Yishun | 5 | 5 | 5 | 5 | 5 | 5 | 5 | 4 | 4 | 5 | 5 | 5 | 5 | 5 | 5 | 6 | 6 | 6 | 6 | 6 | 6 | 6 |
| 158 | Yishun | 4 | 4 | 4 | 4 | 4 | 4 | 4 | 4 | 4 | 4 | 3 | 3 | 4 | 4 | 4 | 4 | 4 | 4 | 4 | 4 | 4 | 4 |
| 161 | Yishun | 6 | 6 | 6 | 6 | 6 | 6 | 6 | 6 | 6 | 6 | 6 | 6 | 6 | 6 | 6 | 6 | 6 | 6 | 6 | 6 | 6 | 6 |
| 162 | Yishun | 6 | 6 | 6 | 6 | 5 | 5 | 6 | 6 | 6 | 6 | 6 | 6 | 6 | 6 | 4 | 4 | 4 | 4 | 6 | 6 | 6 | 6 |
| 296 | Yishun | 6 | 6 | 6 | 6 | 6 | 6 | 6 | 6 | 6 | 6 | 6 | 6 | 6 | 6 | 6 | 6 | 6 | 6 | 6 | 6 | 6 | 6 |

continued Table S4.

TableS5. IBD-based estimates of the dispersal kernel spread (σ) in *Aedes aegypti* from Singapore.

| **TAMPINES** | ***N_e_*** | ***D*** | **genetic distance** | ***b*** | ***N_b_*** | ***σ*** |
| --- | --- | --- | --- | --- | --- | --- |
| method 1 (PWoP) | 863 (863-1112) | 0.0074 (0.0074-0.0095) | PCA | 0.0037 (0.0023-0.0050) | 270 (198-424) | 54.1 (40.8-60.7) |
| method 2 (LDN_e_) | 167 (93-619) | 0.0014 (0.0008-0.0053) |  |  |  | 122.9 (54.7-206.2) |
| method 3 (Gravitrap) | - | 0.0048 (0.0030-0.0066) |  |  |  | 66.8 (48.8-105.6) |
|  |  |  | Rousset *â* | 0.0049 | 204 | 47.0 (41.4-47.0) |
|  |  |  |  |  |  | 106.8 (55.5-106.8) |
|  |  |  |  |  |  | 58.1 (49.5-73.3) |
|  |  |  | Loiselle’s kinship | -0.0039* | 257 | 52.7 (46.4-52.7) |
|  |  |  |  |  |  | 119.8 (62.2-160.5) |
|  |  |  |  |  |  | 65.1 (55.6-82.2) |
| **YISHUN** | ***N_e_*** | ***D*** | **genetic distance** | ***b*** | ***N_b_*** | ***σ*** |
| method 1 (PWoP) | 1185 (1176-1346) | 0.0063 (0.0063-0.0072) | PCA | 0.0065 (0.0051-0.0079) | 154 (127-195) | 44.1(37.6-49.7) |
| method 2 (LDN_e_) | 258 (200-357) | 0.0014 (0.0011-0.0019) |  |  |  | 94.4 (80.3-107.2) |
| method 3 (Gravitrap) | - | 0.0022 (0.0014-0.0030) |  |  |  | 74.4 (63.7-93.3) |
|  |  |  | Rousset *â* | 0.0052 | 192 | 49.2 (46.2-49.4) |
|  |  |  |  |  |  | 105.5 (89.7-119.9) |
|  |  |  |  |  |  | 83.1 (71.2-104.3) |
|  |  |  | Loiselle’s kinship | -0.0058* | 173 | 46.7 (43.8-46.9) |
|  |  |  |  |  |  | 100.1 (85.1-113.7) |
|  |  |  |  |  |  | 78.9 (67.5-98.9) |

The mean (95% CI) for the estimates of: effective population size (*N_e_*), effective density (*D*) calculated using 3 methods (genetic – PWoP, LDN*_e_*; entomological – Gravitrap), IBD slope (*b),* genetic neighborhood size (*N_b_*) calculated from the analysis with different genetic distance measures (PCA, Rousset *â*, Loiselle’s kinship), for data from Tampines and Yishun. *Note the negative relationship between Loiselle’s kinship coefficient and ln-geographic distance (i.e. kinship decreases as the ln-geo distance increases).

TableS6. Mantel and partial Mantel tests for non-close-kin data in Tampines and Yishun.

|  | **Mantel *r* (95%CI)** | ***p*-value** | **temporal distance** | **Partial Mantel *r* (95%CI)** | ***p*-value** |
| --- | --- | --- | --- | --- | --- |
| **TAMPINES** | 0.124 (0.052-0.199) | 0.046 | Weeks apart | N/A | N/A |
|  |  |  | Days apart | 0.151 (0.078-0.240) | 0.01 |
| **YISHUN** | 0.158 (0.112-0.208) | 0.002 | Weeks apart | 0.191 (0.145-0.237) | 0.001 |
|  |  |  | Days apart | 0.173 (0.129-0.225) | 0.002 |

Correlation between genetic and geographic distance matrices (Mantel test, gen dist ~ geo dist), and correlation between genetic and geographic distance matrices once the linear effects of temporal distance are removed (Partial Mantel test, gen dist ~ geo dist + temp dist). Temporal distance was calculated as: the number of weeks or days between the collection dates of the pair members (Weeks apart, Days apart). In Tampines, all non-close kin were sampled in the same week (zero weeks apart), precluding the partial Mantel testing for this case. In all other cases, a positive correlation between genetic and geographic distances remained significant once the effect of temporal distances was removed (p ≤ 0.01), indicating a non-significant contribution of temporal distance to the IBD between non-close kin from Tampines and Yishun.

TableS7. Entomological data used for the estimation of effective population density in *Aedes aegypti* (method 3).

| **Colection** | **No. Females** | | **No. Gravitraps** | | **d_F_** | |
| --- | --- | --- | --- | --- | --- | --- |
| **period** | **T** | **Y** | **T** | **Y** | **T** | **Y** |
| Jan 2018 | 391 | 404 | 1668 | 1296 | 0.0021 | 0.0015 |
| Feb 2018 | 314 | 256 | 1357 | 1048 | 0.0017 | 0.0010 |
| Mar 2018 | 297 | 202 | 1375 | 1044 | 0.0016 | 0.0008 |
| Apr 2018 | 445 | 257 | 1360 | 1050 | 0.0024 | 0.0010 |
| May 2018 | 760 | 280 | 1724 | 1301 | 0.0041 | 0.0011 |

The number of *Aedes aegypti* females collected from Gravitraps that comprised a surveillance system in parts of Tampines (T) and Yishun (Y) from January to May 2018, and were used to estimate the density of females (d_F_).
