# Supplementary material for: Using spatial genetics to quantify mosquito dispersal for control programs
Source: BMC Biol. 2020 Aug 20;18:104. doi: 10.1186/s12915-020-00841-0 (PMC7439557; doi:10.1186/s12915-020-00841-0)

**
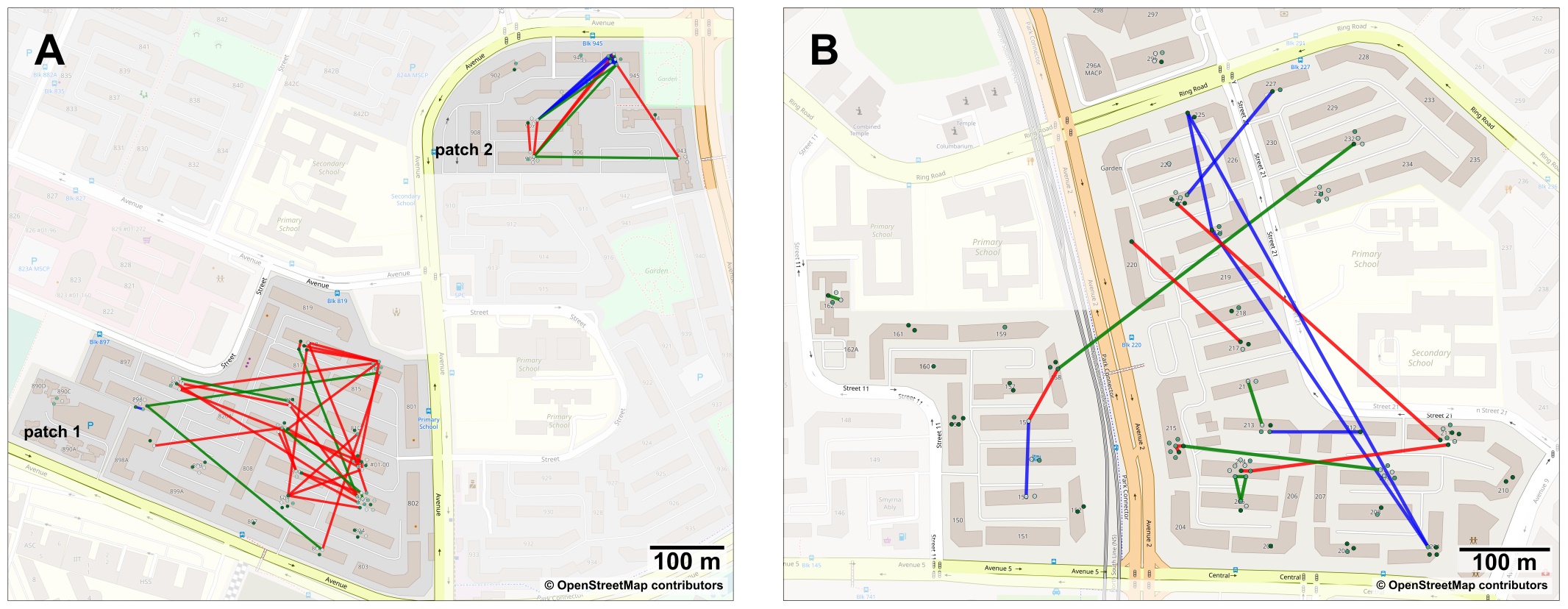
Additional file 2: FigS1. Spatial network of close kin in Tampines (A) and Yishun (B).** Blue lines connect full siblings, green lines connect 2^nd^ degree relatives, and red lines connect 3^rd^ degree relatives. Gravitraps are represented with green dots - one group per building, with shades varying from lighter to darker depending on the altitude (dark green - ground floor, light green - top floor).

**
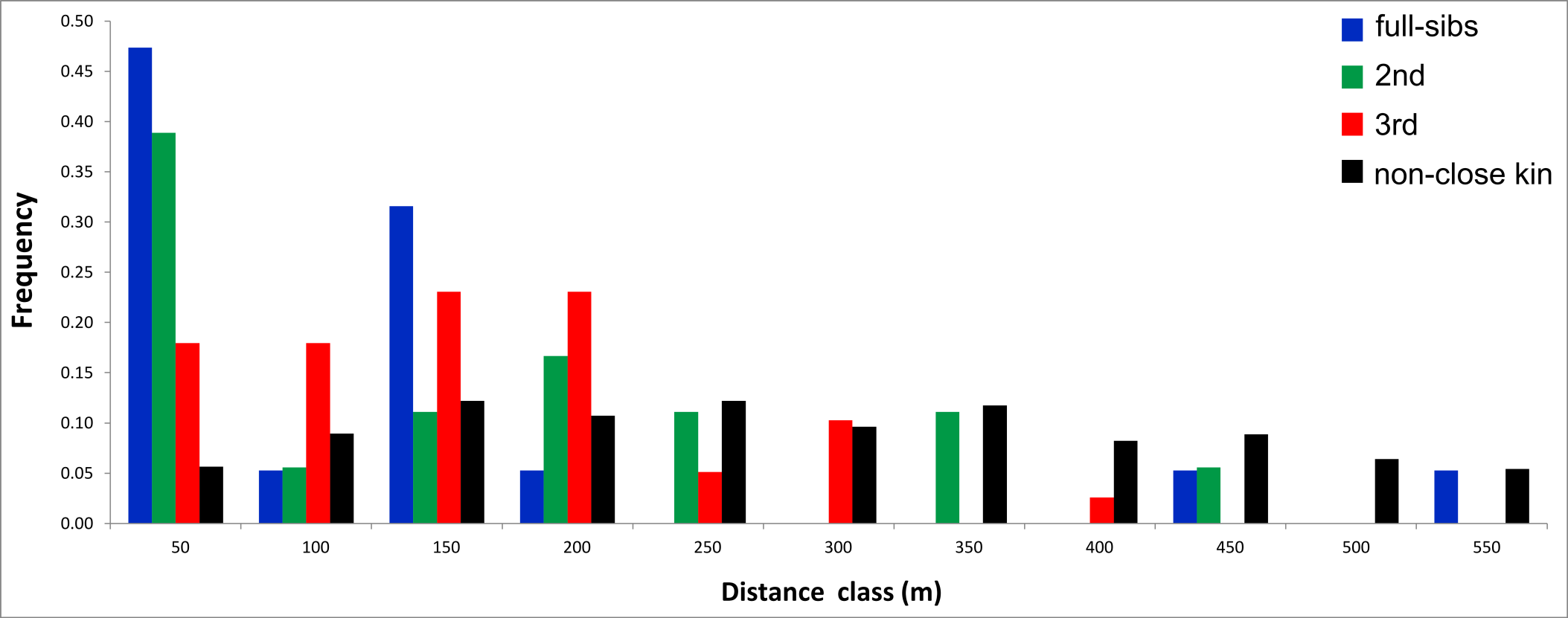
Additional file 2: FigS2. Frequency histogram of separation distance for each kinship category.** Frequency of pairs in each category (blue – full siblings, green – 2nd degree relatives, red – 3rd degree relatives, black – non-close kin) separated by a distance (m) within a 50m-distance category.

**Additional file 2: FigS3. Distribution fitting (dispersal kernel parametrization) analysis.** (A) Q-Q plot for the empirical and theoretical distribution quantiles This goodness-of-the-fit assessment shows a good fit of the empirical to the expected data for Weibull (green) and exponential (red) distributions, but not to the lognormal distribution (blue). (B) Skewness-kurtosis plot. A nonparametric bootstrap procedure (constructed by random sampling with replacement from the empirical data set) was performed to take into account the uncertainty of the estimated values of kurtosis and skewness from data.


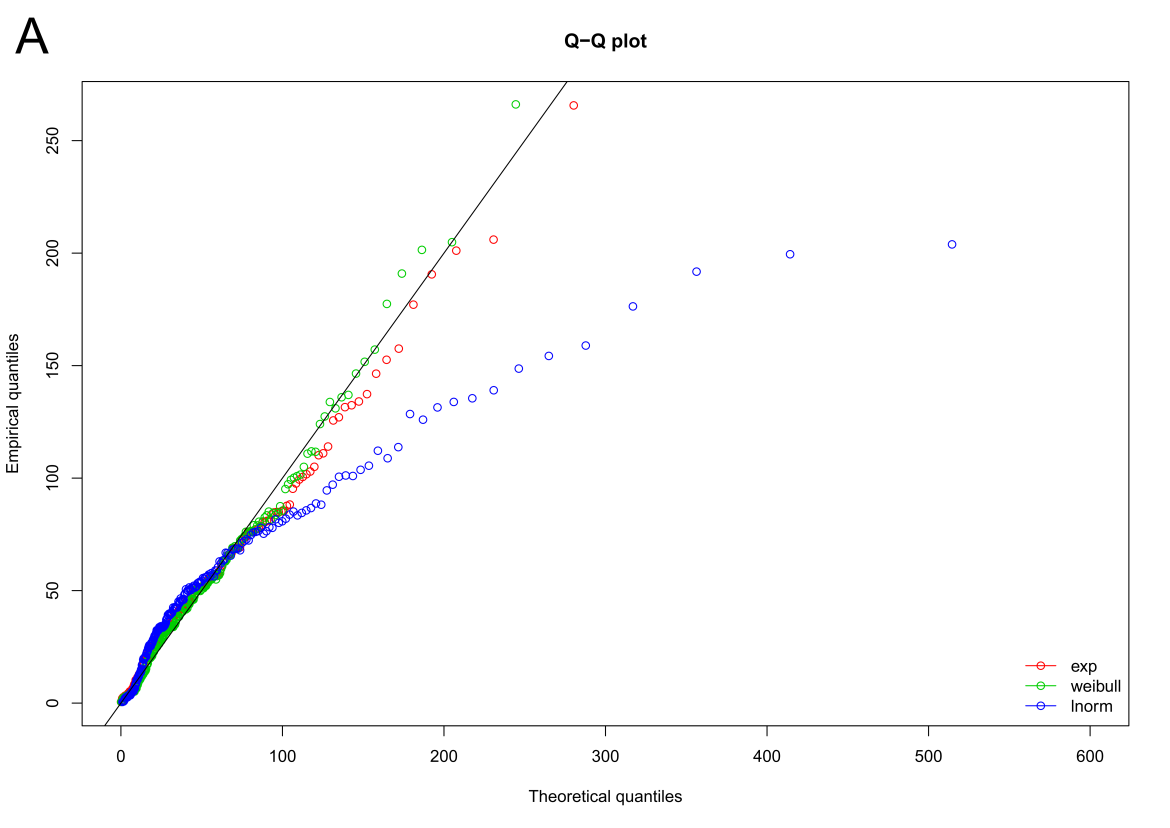

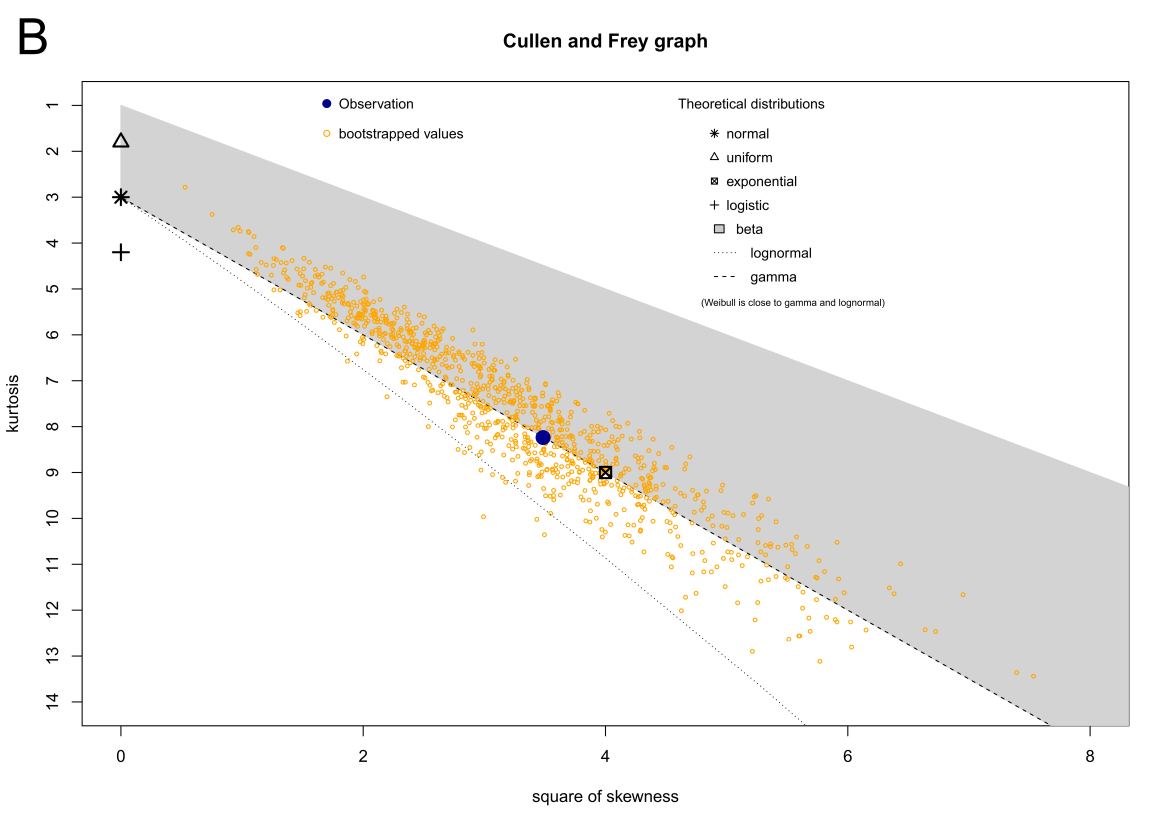

Supplement: Supplementary file 2 — Additional file 2: Fig. S1. Spatial network of close kin in Tampines (A) and Yishun (B). Fig. S2. Frequency histogram of separation distance for each kinship category. Fig. S3. Distribution fitting (dispersal kernel parametrization) analysis. [file 12915_2020_841_MOESM2_ESM.docx]
